# Mitochondrial VDAC1 Silencing Leads to Metabolic Rewiring and the Reprogramming of Tumour Cells into Advanced Differentiated States

**DOI:** 10.3390/cancers10120499

**Published:** 2018-12-08

**Authors:** Tasleem Arif, Avijit Paul, Yakov Krelin, Anna Shteinfer-Kuzmine, Varda Shoshan-Barmatz

**Affiliations:** Department of Life Sciences, The National Institute for Biotechnology in the Negev, Ben-Gurion University of the Negev, Beer-Sheva 84105, Israel; tashu100@gmail.com (T.A.); paulavijitmicro@gmail.com (A.P.); kreliny@gmail.com (Y.K.); shteinfe@post.bgu.ac.il (A.S.-K.)

**Keywords:** cancer stem cells, differentiation, mitochondria, si-RNA, VDAC1

## Abstract

Oncogenic properties, along with the metabolic reprogramming necessary for tumour growth and motility, are acquired by cancer cells. Thus, tumour metabolism is becoming a target for cancer therapy. Here, cancer cell metabolism was tackled by silencing the expression of voltage-dependent anion channel 1 (VDAC1), a mitochondrial protein that controls cell energy, as well as metabolic and survival pathways and that is often over-expressed in many cancers. We demonstrated that silencing VDAC1 expression using human-specific siRNA (si-hVDAC1) inhibited cancer cell growth, both in vitro and in mouse xenograft models of human glioblastoma (U-87MG), lung cancer (A549), and triple negative breast cancer (MDA-MB-231). Importantly, treatment with si-hVDAC1 induced metabolic rewiring of the cancer cells, reversing their oncogenic properties and diverting them towards differentiated-like cells. The si-hVDAC1-treated residual “tumour” showed reprogrammed metabolism, decreased proliferation, inhibited stemness and altered expression of genes and proteins, leading to cell differentiation toward less malignant lineages. These VDAC1 depletion-mediated effects involved alterations in master transcription factors associated with cancer hallmarks, such as highly increased expression of p53 and decreased expression of HIF-1a and c-Myc that regulate signalling pathways (e.g., AMPK, mTOR). High expression of p53 and the pro-apoptotic proteins cytochrome c and caspases without induction of apoptosis points to functions for these proteins in promoting cell differentiation. These results clearly show that VDAC1 depletion similarly leads to a rewiring of cancer cell metabolism in breast and lung cancer and glioblastoma, regardless of origin or mutational status. This metabolic reprogramming results in cell growth arrest and inhibited tumour growth while encouraging cell differentiation, thus generating cells with decreased proliferation capacity. These results further suggest VDAC1 to be an innovative and markedly potent therapeutic target.

## 1. Introduction

Cancer cells typically acquire the same properties, such as self-sufficiency regarding growth signals, unlimited proliferation potential, and resistance to anti-proliferative and apoptotic cues [1]. While different types and etiologies of cancer exist, they frequently present the same bioenergetic abnormalities, regardless of cellular or tissue origin [2]. Warburg first linked metabolism with cancer, viewing cancer as mainly a metabolic disease. Other studies have since enhanced our understanding of how metabolic reprogramming in cancer cells affect tumourigenesis [1,2,3,4]. Tumour cell metabolism is now widely considered a hallmark of cancer, and can even be regarded as an “Achilles’ heel” of the disease, providing a unique therapeutic opportunity to target tumour cells by focusing on their energy metabolism. 

The Warburg effect, also known as aerobic glycolysis, often causes an increase in total glycolysis in solid tumours under both hypoxic conditions and normal oxygen levels [5,6]. Enhanced proliferation brings about increased anabolic needs and, correspondingly, cancer cells rewire their metabolic pathways to divert nutrients like glucose and glutamine into anabolic pathways to meet the demand for cellular building blocks [5,6].

Respiration, oxidative metabolism, and other mitochondrial activities are also required by cancer cells for tumour growth [4,7]. For example, metabolic intermediates generated by the tricarboxylic acid (TCA) cycle fill the role of precursors for lipid, amino acid, and nucleotide biosynthesis. These precursors complement precursor metabolites generated from glycolysis and other pathways and are needed for proliferation. Glutamine can also be an important source of energy [8]. Transformed cells can, moreover, adapt their metabolism in support of tumour initiation and progression, while specific metabolic activities have been thought to enhance cell transformation. These include mutations in genes encoding certain enzymes, such as isocitrate dehydrogenases-1 and -2 (IDH1, IDH2), fumarate hydratase (FH), and proteins of the succinate dehydrogenase (SDH) complex [9].

There are several approaches to target the cell-autonomous metabolic reprogramming of cancer cells. To target glycolysis, 2-deoxyglucose (2-DG) and 3-bromopyruvate (3-BP), an alkylating reagent, have been employed, while lonidamine has been used to disrupt glycolysis and inhibit the activity of hexokinase (HK) [10]. These reagents are not, however, particularly effective (acting at mM levels), and since they do not specifically target cancer cells, they are toxic to certain normal tissues that use glucose as their primary source of energy, such as the brain, testes, and retinae [10]. Furthermore, although 2-DG treatment was effective against tumours, hypoglycemic symptoms also appeared [11]. The pyruvate dehydrogenase kinase (PDHK) inhibitor dichloroacetate (DCA) produces metabolic effects in human tumours [12], although limited efficacy data have been reported. Drugs for mutant IDH have some impact on patients with haematological malignancies, yet they are less effective in models of glioma [13].

The voltage-dependent anion channel (VDAC1), serving as the mitochondria gatekeeper, can equally be considered as a target in tackling the altered metabolism of cancer cells. Found at the outer mitochondrial membrane (OMM), VDAC1 provides the main transport pathway for metabolites, including pyruvate, malate, succinate, nucleotides and NADH, and is also involved in cholesterol and lipid transport and mediate the fluxes of ions, such as Ca^2+^ [14,15,16]. VDAC1 plays a role in mitochondria-ER Ca^2+^ signalling, serves as the ROS transporter and regulates mitochondrial and cytosol redox states [14,15,16]. As a transporter of metabolites, VDAC1 controls cell energy and metabolic homeostasis, is over-expressed in many cancers [16,17] and contributes to the metabolic phenotype of cancer cells. 

The positioning of VDAC1 at the OMM facilitates its interactions with proteins that mediate and regulate the integration of mitochondrial functions with other cellular activities. For instance, VDAC1 forms a complex with HK, the adenine nucleotide translocase (ANT), and creatine kinase [14,15,16]. The interaction of VDAC1 with HK allows for coupling between oxidative phosphorylation (OXPHOS) and glycolysis, an important factor in cancer cell energy homeostasis (i.e., the Warburg effect). VDAC1 is also implicated in apoptosis, participating in the release of apoptotic factors from mitochondria and interacting with anti-apoptotic regulators [14,16,18]. Thus, VDAC1 appears to be a convergence point for a variety of cell survival and death signals, mediated through its association with various ligands and proteins.

VDAC1 is highly expressed in various tumours [16,17], which points to its crucial role in the metabolic and survival pathways of cancer cells. This concept is further backed by studies showing that down-regulation of VDAC1 expression led to reduced metabolite exchange between mitochondria and the cytosol and inhibition of the growth of various cancer cell types and tumours [17,19,20]. Recently, we used glioblastoma cell lines in culture as well as in sub-cutaneous and intracranial-orthotopic glioblastoma (GBM) models, and demonstrated that silencing the expression of mitochondrial VDAC1 using specific si-RNA resulted in a rewiring of cancer cell metabolism, stemness, and induced differentiation into neuronal-like cells [21].

In this study, we asked whether si-hVDAC1 would similarly affect other cancers with respect to reprogramming metabolism, reduced cancer stem cell (CSC) levels and induced differentiation. For this, we addressed breast cancer (MDA-MB-231), lung cancer (A549) and GBM (U-87MG) cell lines and tested the effects of si-hVDAC1 in cell culture and sub-cutaneous mouse models.

We demonstrate that in the three selected cancers, regardless of their different origin and mutations carried, treatment with si-hVDAC1 altered cancer hallmarks, including a rewiring of cell metabolism and of pathways for growth and survival at the root of the malignant phenotype. These include inhibiting cell proliferation and tumour growth, eliminating cancer stem cells, and inducing cell maturation/differentiation. The results support emerging paradigms that cancer cell metabolism controls cancer hallmarks and that its reprogramming reverses tumour properties, independent of the mutations carried. In addition, down-regulation of VDAC1 function as the mitochondrial gatekeeper, rather than attacking metabolism via targeting the metabolism of a specific enzyme, is a promising strategy to treat cancer. 

## 2. Methods 

### 2.1. Materials 

We obtained the cell transfection agents JetPRIME and JetPEI from PolyPlus transfection (Illkirch, France), while we obtained non-modified and 2′-O-methyl-modified hVDAC1-siRNAs from Genepharma (Suzhou, China). We obtained bovine serum albumin (BSA), β-mercaptoethanol, phenylmethylsulfonyl fluoride (PMSF), propidium iodide (PI), adenosine triphosphate (ATP), carbonyl cyanide-*p*-trifluoromethoxyphenyl hydrazone (FCCP), Sulforhodamine B (SRB), Triton X-100, Tween-20, tetramethylrhodamine methyl ester (TMRM), hematoxylin, and eosin from Sigma (St. Louis, MO, USA). We obtained a TUNEL assay kit from Promega (Madison, WI, USA) and paraformaldehyde from Emsdiasum (Hatfield, PA, USA). Dulbecco’s modified Eagle’s medium (DMEM) was obtained from Gibco (Grand Island, NY, USA). Fetal calf serum (FCS), normal goat serum (NGS), and penicillin-streptomycin was obtained from Biological Industries (Beit Haemek, Israel). Primary antibodies, their sources and the dilutions used are laid out in Supplementary Table S1. Horseradish peroxidase (HRP)-conjugated anti-mouse as well as anti-rabbit and anti-goat antibodies were from KPL (Gaithersburg, MD, USA). We obtained 3,3-diaminobenzidine (DAB) from (ImmPact-DAB, Burlingame, CA, USA) and procured tissue array slides from Biomax (US Biomax, Derwood, MD, USA). 

### 2.2. Cell Culture and Transfection

U-87MG (human glioblastoma), A549 (non-small lung carcinoma), MDA-MB-231 (human breast carcinoma), HaCaT (spontaneously immortalised aneuploid keratinocyte cell line from adult human skin) and WI-38 (human lung fibroblast-derived), cells were obtained from ATCC and maintained at 37 °C and 5% CO_2_ in the recommended culture medium and supplements. Mycoplasmal contamination in all cell lines was routinely tested using PCR and appropriate primes according to the manufacturer’s instructions (Biological Industries, Beit-Haemek, Israel). 2′-O-methyl-modified hVDAC1-siRNAs were synthesised by Genepharma. The following sequences were used, with 2′-O-methyl-modified nucleotides highlighted in bold and underline (nucleotide positions are provided for sense (S) and anti-sense (AS) sequences): si-hVDAC12/A, S: 238-5′ACAC**U**AG**G**CACC**G**AGA**U**UA3′-256 and AS: 238-5′UAAUC**U**CGGUGCCU A**G**UGU3′. Cells were seeded (150,000 cells/well) in 6-well culture dishes to 40–60% confluence and transfected with 10–100 nM si-NT or si-hVDAC1 using the JetPRIME transfection reagent, according to the manufacturer’s instructions. U-87MG, A549 and MDA-MB-231 cells were transiently transfected with plasmid pcDNA4/TO (2 μg DNA) encoding native murine (m)VDAC1 using the JetPRIME reagent according to the manufacturer’s instructions.

### 2.3. SRB Assay for Cell Proliferation, Mitochondrial Membrane Potential, Cellular ATP Levels Determination 

Twenty-four hours post-transfection with si-NT or si-hVDAC1, A549, MDA-MB-231, U-87MG, WI-38, and HaCaT cells were subsequently subjected to a cell proliferation assay (SRB), mitochondrial membrane potential (ΔΨ) assessment and cellular ATP levels determination, as previously described [21].

### 2.4. Xenograft Experiments

U-87MG (3 × 10^6^), A549 (5 × 10^6^) and MDA-MB-231 (3 × 10^6^) cells were subcutaneously (s.c.) inoculated into the hind leg flanks of athymic eight-week-old male nude mice (Envigo, Israel). Fourteen days after inoculation, the volume of the tumour was measured (50–100 mm^3^) and the mice were randomised into two groups (3–6 animals/group), treated with si-NT or si-hVDAC1 mixed with in vivo JetPEI reagent and injected into the established s.c. tumours (50 nM final, 2 boluses) once every three days. At the end of the experiment, the mice were sacrificed, tumours were excised and half of each tumour was either fixed and processed for immunohistochemistry (IHC) or frozen in liquid nitrogen for later immunoblot and RNA isolation. Approval for the experimental protocol was obtained from the Institutional Animal Care and Use Committee of the Soroka University Medical Center.

### 2.5. IHC and Immunofluorescence (IF), Immunoblotting and TUNEL Assays

Immunohistochemical, immunofluorescent or TUNEL staining was performed on 5 μm-thick formalin-fixed and paraffin-embedded tumour tissue sections. Immunoblotting of tumour tissues extracts with the antibodies indicated (Supplementary Table S2) were carried out as described [21]. Quantitative analysis of the IHC images was carried out using a panoramic scanner (panoramic MIDI II, 3DHISTH) and HistoQuant software.

### 2.6. RNA Preparation and Quantitative Real-Time PCR (q-RT-PCR) 

Total RNA was isolated from si-NT-treated tumours (TTs), and si-hVDAC1-TTs (from 3–4 mice each) and used for complementary DNA synthesis followed by real-time RT-PCR using specific primers (Table S3) as previously described [21]. Copy numbers for each sample were calculated by the CT-based calibrated standard curve approach. The mean fold change (± SEM) of the three replicates of three independent assays was calculated. 

### 2.7. Statistics

Means ± SEM of results from independent experiments are shown. A difference was taken as statistically significant when the *p*-value was ≤ 0.05 (*), ≤ 0.01 (**), ≤0.001 (***), ≤ 0.0001 (****). 

## 3. Results 

We have shown in a previous study with GBM that silencing VDAC1 expression in tumours led to cancer-reprogrammed metabolism [21]. Tumour treatment with si-RNA against human (h) VDAC1 resulted in rewired metabolism, inhibited cell proliferation, epithelial-mesenchymal transition (EMT), invasion, angiogenesis and stemness, while leading to tumour cell differentiation into neuronal-like cells [21]. Here, we asked whether such interplay between oncogenic signalling networks and metabolism resulting in a multi-pronged attack on cancer hallmarks is common to other cancers, such as breast and lung cancers that differ in their origin and mutations carried (Table S1). For this, the MDA-MB-231, A549 and U-87MG cell lines were used. 

MDA-MB-231 cells are an invasive, aggressive, poorly differentiated breast adenocarcinoma cell line derived from a pleural effusion metastatic tumour. These cells are defined as triple negative breast cancer (TNBC) due to the absence of estrogen receptors (ER), progesterone receptors (PR), and human epidermal growth factor receptor-2 (HER2). MDA-MB-231 cells carry mutations, such as those in the *BRAF, RAS, CDKN2A, TP53, PTEN, BRIP1* and *LIFR* genes. The features associated with mammary CSCs are defined by CD44^+^ and CD24^−^/low phenotype [22]. 

A549 cells are from a non-small cell lung carcinoma (NSLC) cell line derived from a primary tumour. A549 cells are characterised as pre-alveolar type II pneumocytes of the human lung due to the expression of high numbers of multilamellar bodies [23]. A549 cells also carry several mutated genes associated with tumourigenicity, such as those in the *RAS, CDKN2A, FLT3, CBL, KEAP1, ZFHX3, FH, FUS, STK11, ATR, SUFU, HIP1* and *SMARCA4*. 

U-87MG cells are from glioblastoma hypodiploid cells derived from a primary brain tumour. U-87MG cells carry several mutations associated with tumourigenicity, including those in *CDKN2A, RAS, PTEN, HF1* and *PCM1*. 

### 3.1. Silencing VDAC1 Expression Inhibits Cancer Cell Growth and Tumour Development

**Figure 1:**
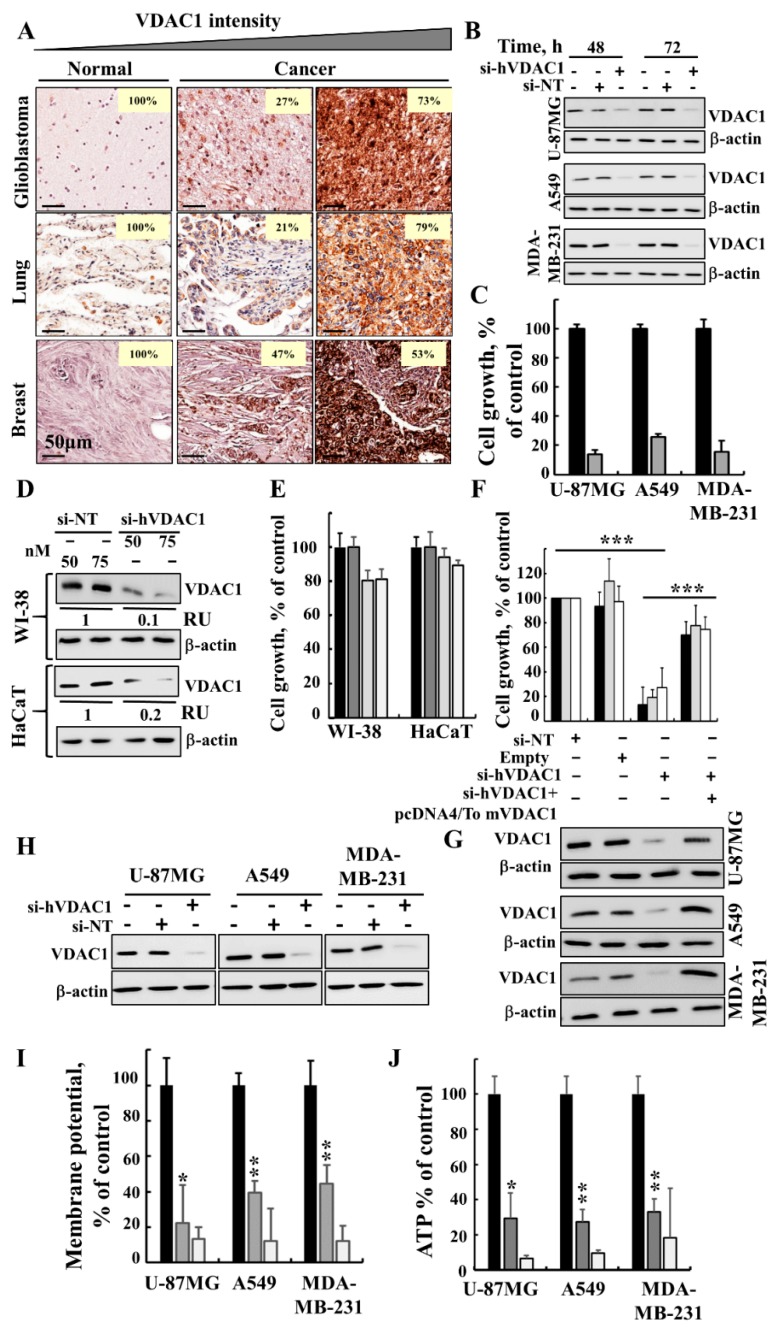
si-hVDAC1 treatment silences VDAC1 expression, causes cell growth inhibition and reduces energy production. (**A**) IHC staining of VDAC1 in sections derived from human normal tissue (*n* = 13), glioblastoma (*n* = 40), lung cancer (*n* = 20) and breast cancer (*n* = 20) in tissue microarray slides (Biomax). Percentages of sections stained at the indicated intensity are shown. (**B**, **C**) U-87MG, A549 and MDA-MB-231 cells were treated with 50 nM si-NT (black bars) or si-hVDAC1 (grey bars) and 72 h post-treatment were analysed for VDAC1 levels by immunoblotting (**B**) and cell growth using the SRB assay (mean ± SEM; *n* = 3) (**C**). (**D**, **E**) WI-38 and HaCaT cells treated with si-NT (50 or 75 nM, black and grey bars, respectively) or si-hVDAC1 (50 or 75 nM, light grey and white bars, respectively) and analysed for VDAC1 levels by immunoblotting 48 h post-transfection (RU indicates relative value) (**D**) and for cell growth using the SRB assay (mean ± SEM; *n* = 3) (**E**). **F**, **G**) U-87MG (black bars), A549 (light grey bars) and MDA-MB-231 cells (white bars) were transfected with si-NT or si-hVDAC1 (50 nM) and 24 h post-transfection, the cells were again transfected with plasmid pcDNA4/TO, either empty or encoding mVDAC1. After 24 h, cell growth was analysed by the SRB method (mean ± SEM; *n* = 3) (**F**) or analysed for VDAC1 levels by immunoblotting (**G**). (**H**–**J**) Immunoblot (**H**), mitochondrial membrane potential **(**ΔΨ) (**I**) and ATP (**J**) levels were analysed in U-87MG, A549 and MDA-MB-231 cells treated with 50 nM si-NT (black bars) or si-hVDAC1 (grey bars). Cells treated with FCCP, (25 μM) (white bars) served as controls for decreasing ΔΨ and ATP levels. β-actin served as an internal loading control. Mean ± SEM; *n* = 3; * *p* ≤ 0.05; ** *p* ≤ 0.01; *** *p* ≤ 0.001.

As in GBM [21], VDAC1 is also over-expressed in patient-derived lung and breast cancer samples (Figure 1A). Silencing VDAC1 expression in U-87MG, A549 and MDA-MB-231 cells by si-hVDAC1 led to marked decreases in both VDAC1 levels (80–90%) (Figure 1B) and cell growth (Figure 1C). Non-targeting siRNA (si-NT) had no significant effect on VDAC1 expression levels or cell growth (Figure 1B,C). In addition, in non-cancerous immortalised cells, such as WI-38 and HaCaT cells, si-hVDAC1 decreased VDAC1 expression, yet only slightly inhibited cell growth (Figure 1D,E). As the hVDAC1 siRNA sequence used (nucleotides 238–256) differs from the corresponding murine VDAC1 (mVDAC1) sequence by four nucleotides, it reduced VDAC1 expression in human but not murine cells [17,21]. Thus, we were able to restore the reduced cell growth in the three cancer cell lines, U-87MG, A549 and MDA-MB-231, resulting from hVDAC1 silencing by expression of mVDAC1 (Figure 1F,G), pointing to the specificity of the si-hVDAC1 used.

Finally, reduced hVDAC1 levels are expected to limit nutrient and metabolite traffic across the OMM, [19]. Indeed, this was reflected in the reduced mitochondrial membrane potential (ΔΨ) and cellular ATP levels in the si-hVDAC1-treated cells (Figure 1H–J), leading to cell growth inhibition. 

**Figure 2:**
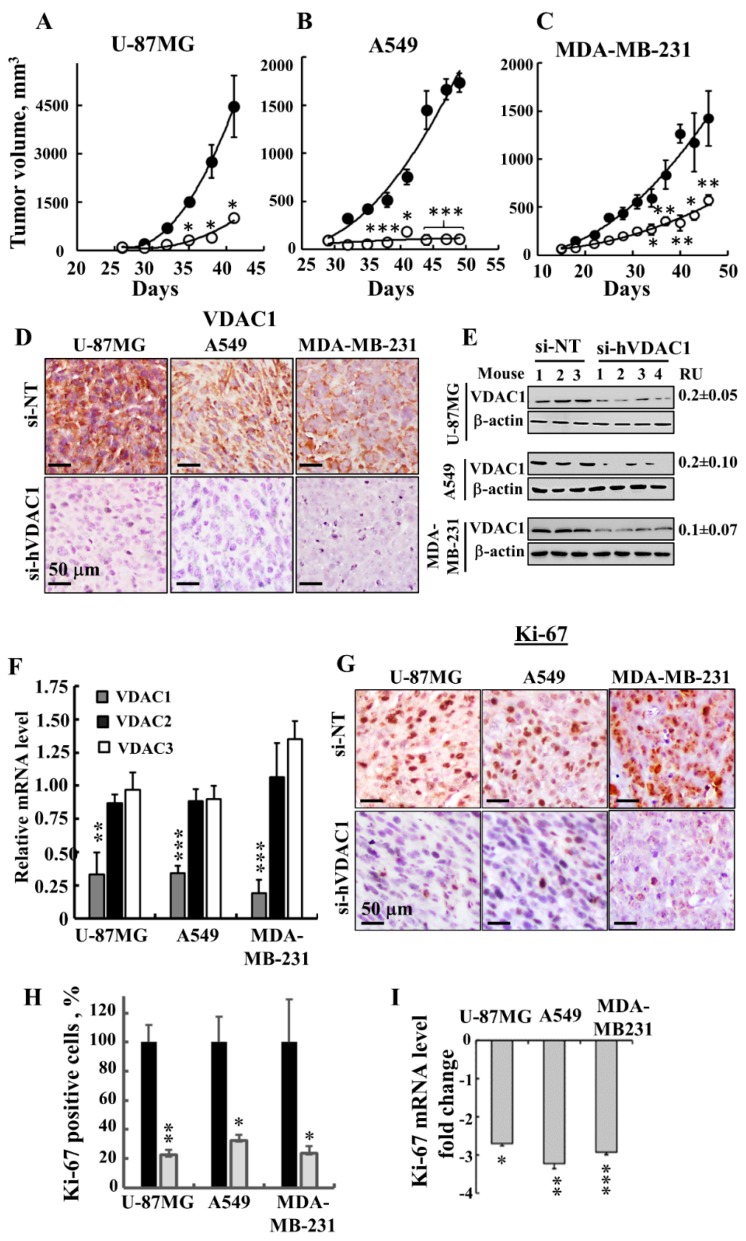
si-hVDAC1 inhibits GBM-, A549 lung cancer- and MDA-MB-231 breast cancer-derived tumour growth in a xenograft mouse model. (**A**–**C**) U-87MG (**A**), A549 (**B**) and MDA-MB-231 (**C**) cells were subcutaneously inoculated into nude mice. When tumour size reached 50-100 mm^3^, the mice were divided into 2 matched groups and xenografts were injected intratumourally every 3 days with si-NT (•, 4–5 mice) or si-hVDAC1 (○, 3–6 mice) to a final concentration of 50–60 nM. The calculated average tumour volumes are presented as means ± SEM. (**D**, **E**) si-NT-TT and si-hVDAC1-TT sections from U-87MG, A549 and MDA-MB-231 xenograft mice were stained for VDAC1 by IHC (**D**) or subjected to immunoblot (**E**). RU = average relative units, presented as the mean ± SEM; *n* = 3–4 mice. β-actin served as an internal loading control. (**F**) Levels of VDAC1, VDAC2 and VDAC3 mRNA in si-hVDAC1-TTs from U-87MG, A549 and MDA-MB-231 cells, as analysed by q-RT-PCR and presented relative to the levels seen in si-NT-TTs. (**G**) Representative IHC staining of si-NT-TTs and si-hVDAC1-TTs derived from U-87MG-, A549- and MDA-MB-231 cells with anti-Ki-67 antibodies. (**H**, **I**) Quantitative analysis of Ki-67-positive cells (**H**) and Ki-67 mRNA levels (**I**) in U-87MG-, A549- and MDA-MB-231-derived tumours presented as fold of decrease. Results show the mean ± SEM (*n* = 3–5), *p*: * ≤ 0.05; ** ≤ 0.01, *** ≤ 0.001.

Next, the effects of si-hVDAC1 on U-87MG-, A549- and MDA-MB-231-derived s.c. tumour xenografts established in athymic nude mice were tested (Figure 2). After the development of a tumour, we separated the mice into two matched groups, injected them intratumourally every 3 days with si-NT or si-hVDAC1 to a final concentration of 50 nM, and followed their tumour growth. In si-NT-injected tumours, tumour volume was increased 71-, 18- and 22-fold for U-87MG, A549 and MDA-MB-231 cells, respectively. However, the growth of si-hVDAC1-TTs was markedly inhibited, with about 94%, 77% and 60% inhibition seen with A549, U-87MG and MDA-MB-231 cells, respectively (Figure 2A–C). 

After the mice were sacrificed, the tumours were excised and frozen or fixed in formalin and sections were IHC-stained for VDAC1 expression. si-NT-TTs were strongly immuno-stained, while, as expected, si-hVDAC1-TT staining was very weak (Figure 2D). Similar results were obtained by immunoblotting (Figure 2E), where decreases in VDAC1 levels (80–90%) were noted. 

Analysis of the three VDAC isoforms using q-RT-PCR revealed that the levels of VDAC1 in si-hVDAC1-TTs, relative to those levels in si-NT-TTs, were decreased by 67, 66 and 81% in U-87MG, MDA-MB-231 and A549 cells, respectively, while only a 10–20% decrease in VDAC2 and VDAC3 mRNA levels was obtained (Figure 2F). si-hVDAC1 tumour treatment also decreased the expression of the cell proliferation marker Ki-67, as shown by IHC staining and quantitative analysis (70–80%) or by q-RT-PCR analysis (2.8 to 3.1-fold) (Figure 2G–I).

DNA microarray analysis of U-87MG-derived tumours showed that cell cycle-related genes were differentially expressed between si-NT-TTs and si-hVDAC1-TTs (Table 1). These results together demonstrate that VDAC1 depletion caused an inhibition of cell proliferation and tumour growth in the three cell line-derived tumours.

### 3.2. Reprogrammed Cancer Cell Metabolism is Reversed by VDAC1 Depletion 

**Figure 3:**
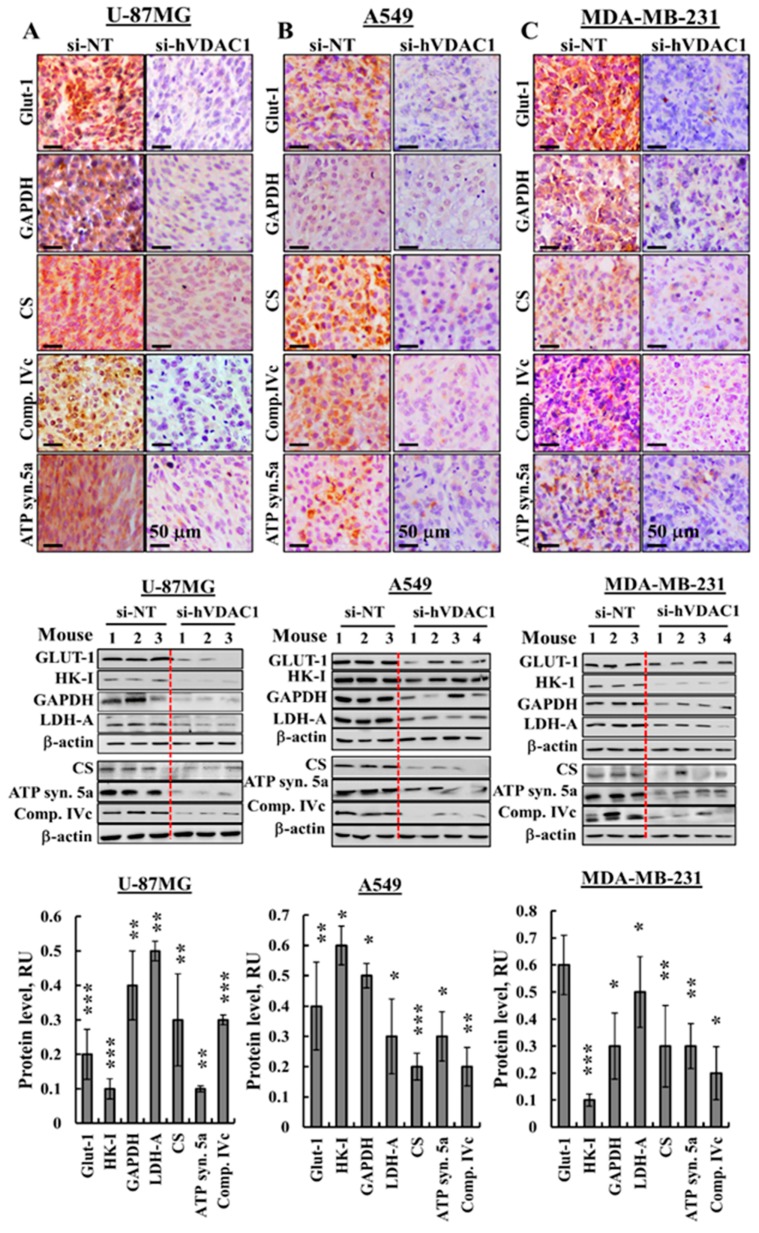

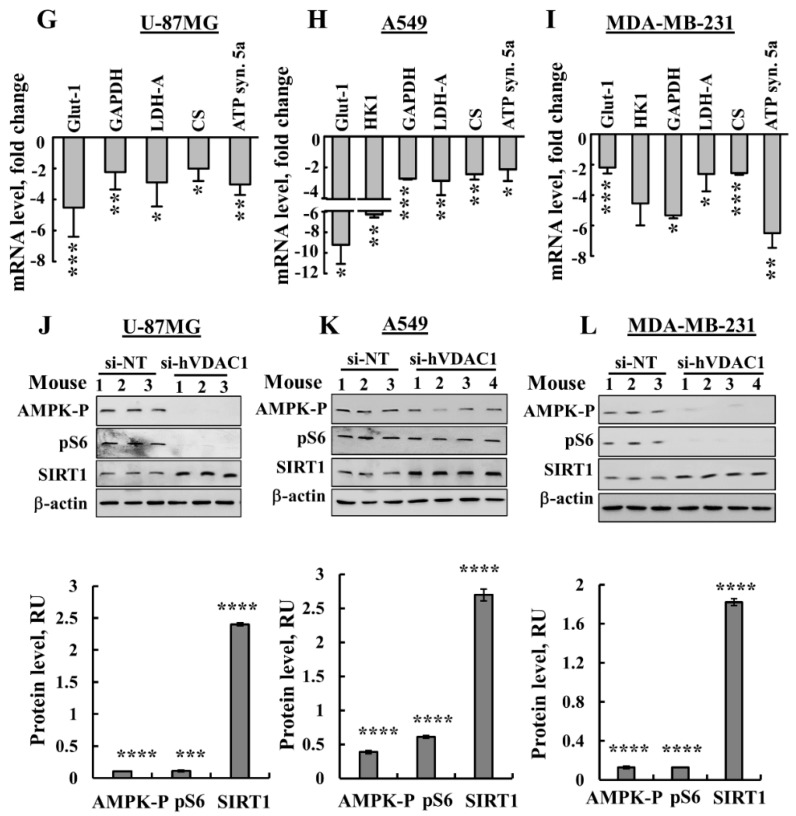
si-hVDAC1 treatment reverses the reprogrammed metabolism of U-87MG-, A549- and MDA-MB-231-derived tumours. (**A**–**C**) Representative IHC staining using specific antibodies against Glut-1, GAPDH, CS, complex IVc (comp. IVc) and ATP synthase 5a (ATP syn. 5a) from si-NT-TTs or si-hVDAC1-TTs sections derived from U-87MG (**A**), A549 (**B**) and MDA-MB-231 (**C**) xenografts. (**D**–**F**) Immunoblots and quantitative analysis of selected metabolism-related proteins from si-NT-TT or si-hVDAC1-TT sections from U-87MG (**D**), A549 (**E**) and MDA-MB-231 (**F**) xenografts. Quantitative analysis is presented as the mean ± SEM in relative units (RU), (*n* = 3–4 mice). β-actin served as an internal loading control. (**G**–**I**) mRNA levels of metabolic enzymes in si-hVDAC1-TTs, relative to those in si-NT-TTs derived from U-87MG (**G**), A549 (**H**) and MDA-MB-231 (**I**) tumours, represented as fold change. Results are means ± SEM (*n* = 5 tumours for each), *p*: * ≤ 0.05; ** ≤ 0.001; *** ≤ 0.0001. (**J**–**L**) Immunoblots and quantitative analysis of phosphorylated AMPK, phospho-S6 (pS6) and SIRT1 using specific antibodies in si-hVDAC1-TTs and si-NT-TTs derived from U-87MG (**J**), A549 (**K**) and MDA-MB-231 (**L**) cells. RU, relative units; the mean ± SEM (*n* = 3–4 mice) are shown, *p*: * ≤ 0.05; ** ≤ 0.01; *** ≤ 0.001, **** ≤ 0.0001; β-actin as an internal loading control are shown.

The metabolic alterations seen during malignant transformation involve a spectrum of functional aberrations and mutations which contribute to elevated glycolysis and increased expression levels of glucose transporters (Glut-1) and glycolytic enzymes [24] (Figure 3). IHC of si-hVDAC1-TTs derived from U-87MG, A549 or MDA-MB-231 cells showed dramatic decreases of Glut-1 and glyceraldehyde dehydrogenase (GAPDH) levels, as compared to si-NT-TTs (Figure 3A–C, Figure S1A). Similar results were obtained for the above proteins and hexokinase (HK-I) and lactate dehydrogenase-A (LDH-A) by immunoblotting (Figure 3D–F) and q-RT-PCR (Figure 3G–I). Expression levels of the Kreb’s cycle enzyme citrate synthase (CS), the subunits of mitochondrial electron transport complex. IVc, and ATP synthase 5a were also highly reduced in si-hVDAC1-TTs, as analysed by IHC (Figure 3A–C, Figure S1A), immunoblotting (Figure 3D–F) or q-RT-PCR (Figure 3G–I), consistent with alterations in OXPHOS. The decreased expression of Kreb’s cycle and OXPHOS enzymes also agrees with the concept that cancer cells combine glycolysis and mitochondria to produce energy, reflecting prevalent normoxic or hypoxic conditions [4,25].

Next, we analysed the effects of si-hVDAC1 on several metabolism regulatory proteins. The AMP-activated protein kinase (AMPK), which was proposed to have both pro- and anti-tumourigenic properties, is a central regulator of cellular metabolism, energy and redox homeostasis under various metabolic stress conditions [26,27]. Antibodies specific to activated phosphorylated AMPK (pAMPK) indicated the presence of high levels of this version of the enzyme in the si-NT-TTs, whereas almost no pAMPK was detected in the si-h-VDAC1-TTs derived from the three cancer cell lines (Figure 3J–L). 

Mammalian target of rapamycin (mTOR) functions as a serine/threonine protein kinase regulating cell proliferation and growth by integrating signals arising from nutrients, growth factors, and energy status [28]. 

Cancer cell growth and survival are associated with mTOR signalling activity [29]. si-hVDAC1-TTs showed low levels of phosphorylated ribosomal S6 (pS6), commonly used as a marker for neuronal activity and a readout of mammalian target of mTORC1 [30] in the three cells derived-si-hVDAC1-TTs (Figure 3J–L). Finally, sirtuin 1 (SIRT1), which deacylates histones and non-histone proteins, was increased in si-hVDAC1-TTs derived from the three cancer types (Figure 3J–L). Taken together, these findings indicate a similar reversal of the metabolic reprogramming of cancer cells in the three tumour types that were tested upon silencing VDAC1 expression.

### 3.3. Treating Tumours with si-hVDAC1 Eliminates CSCs

**Figure 4:**
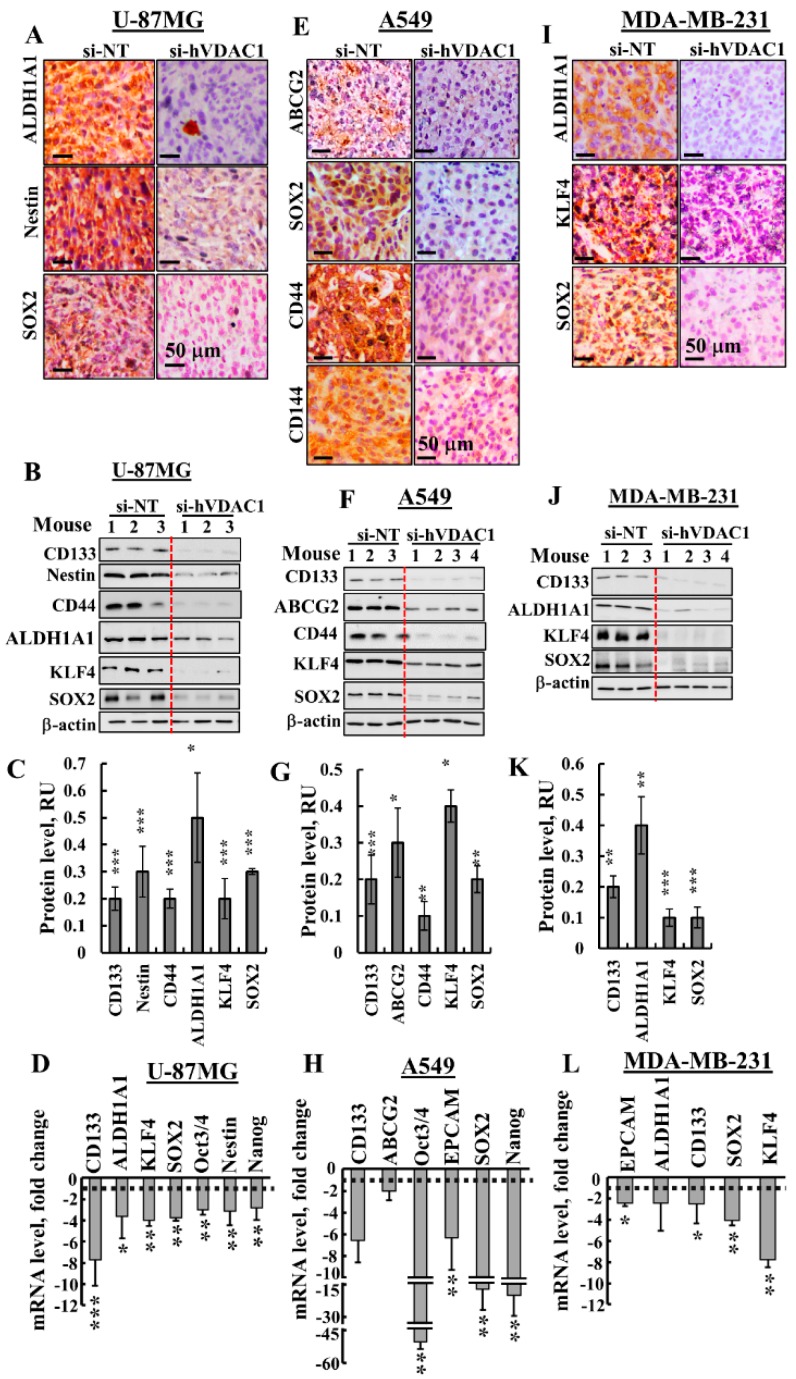
si-hVDAC1 treatment markedly reduces cancer stem cell marker expression in U-87MG-, A549- and MDA-MB-231-derived tumours. Representative IHC staining with cell line-specific CSC markers using specific antibodies in si-NT-TT or si-hVDAC1-TT sections derived from U-87MG (**A**), A549 (**E,**) and MDA-MB-231 (**I**) xenografts. Immunoblot of protein extracts obtained from si-NT-TTs or si-hVDAC1-TTs derived from U-87MG (**B**, **C**), A549 (**F**, **G**) and MDA-MB-231 (**J**, **K**) xenografts, using the specific antibodies indicated, and their quantitative analysis presented as relative units (RU); Results are the mean ± SEM (*n* = 3–4 mice) are shown. β-actin served as an internal loading control. mRNA levels of the indicated genes in si-hVDAC1-TTs relative to those in si-NT-TTs derived from U-87MG (**D**), A549 (**H**) and MDA-MB-231 (**L**) tumours. Results are means ± SEM (*n* = 3–5 tumours), *p*: * ≤ 0.05; ** ≤ 0.01; *** ≤ 0.001. Dashed lines indicate the control level.

The effects of VDAC1 silencing on CSCs were analysed by following the expression of CSC-associated markers specific to U-87MG, A549 and MDA-MB-231 cells (Figure 4). In U-87MG si-hVDAC1-TTs, the expression of CSC markers, such as aldehyde dehydrogenase isoform 1 (ALD1HA1), Nestin, SOX2, CD133, CD44, KLF4, Oct3/4 and Nanog, as evaluated by IHC, immunoblotting or q-PCR, were markedly decreased (Figure 4A–D, Figure S1B). Similarly, si-hVDAC1 treatment of A549-derived tumours greatly decreased the expression of ABCG2, SOX2, CD44, CD144, CD133, KLF4, Oct3/4, EPCAM and Nanog, also as evaluated by IHC, immunoblotting or q-RT-PCR (Figure 4E–H, Figure S1B). In the case of MDA-MB-231-derived tumours treated with si-hVDAC1, the expression of ALD1HA1, KLF4, SOX2, CD133 and EPCAM were highly reduced, again as analysed by IHC, immunoblotting or q-RT-PCR (Figure 4I–L, Figure S1B). 

The q-RT-PCR results demonstrated that the effects of si-hVDAC1 on the expression of the CSC transcription factors Oct3/4, SOX2 and Nanog were highest in A549-derived tumours, where expression levels were decreased 15–45-fold (Figure 4H). In U-87MG and MDA-MB-231-derived tumours, the decrease in their expression was about 3–4-fold (Figure 4D,L). This trend correlates with the highest decrease in tumour size seen with the A549-derived tumour (94%, in comparison to 77% and 60% decreases in the sizes of U-87MG- and MDA-MB-231-derived tumours, as induced by si-hVDAC1, respectively) (Figure 2A–C).

The strong decrease in the levels of CSC markers in the different cells seen upon reducing VDAC1 levels suggests a reduction in CSC levels upon metabolic reprogramming that may result from CSCs differentiation. 

### 3.4. Differentiation of Cancer Cells in GBM, Lung Cancer and Breast Cancer Tumours

**Figure 5:**
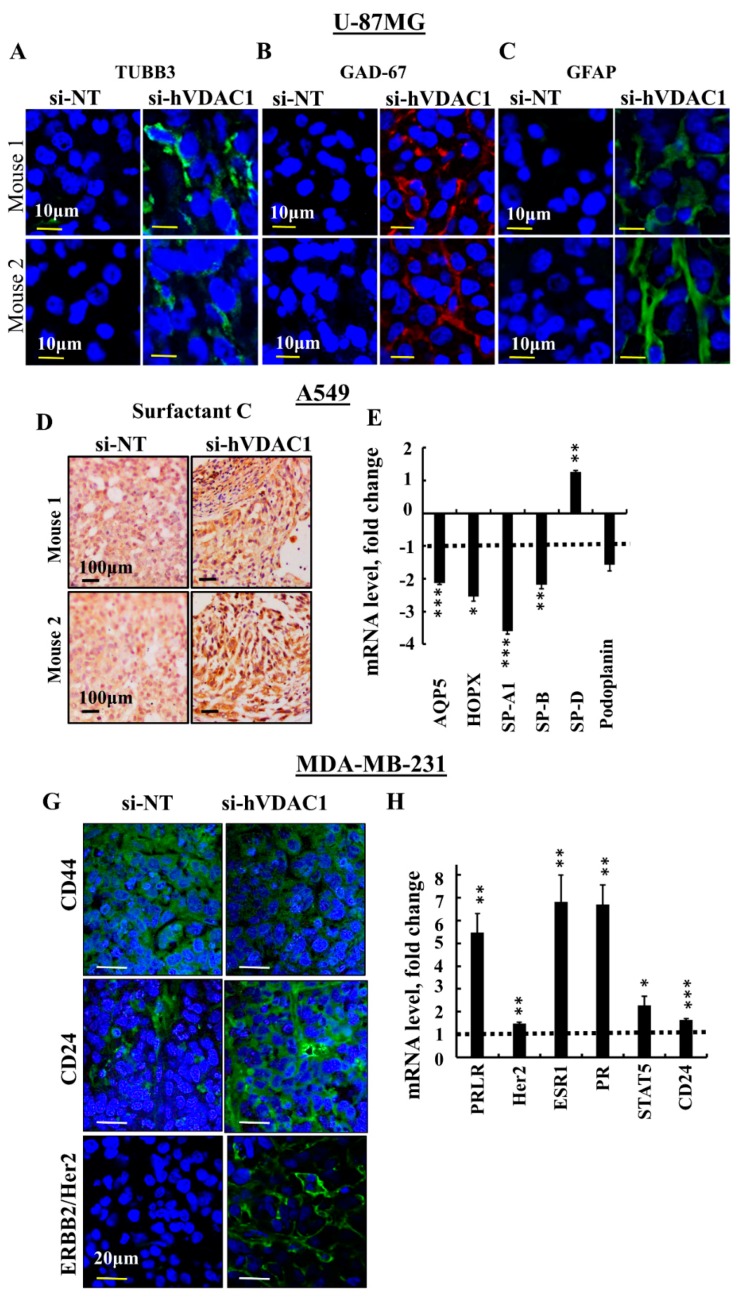
si-hVDAC1 treatment of U-87MG-, A549- and MDA-MB-231-derived tumours induces expression of proteins associated with differentiation. U-87MG cells-derived hVDAC1-TTs or si-NT-TTs were stained with anti-TUBB3 (**A**) anti-GAD-67 (**B**) or anti-GFAP antibodies (**C**) with representative images from 2 mice each shown. A549 cell-derived hVDAC1-TTs or si-NT-TTs were stained for surfactant C (**D**) or subjected to q-RT-PCR to analyse expression levels of the indicated genes (**E**). MDA-MB-231 cell-derived hVDAC1-TTs or si-NT-TTs were stained with anti-CD44, -CD24 or -ERBB2/Her2 antibodies (**G**). mRNA levels of the indicated genes were analysed in si-hVDAC1-TTs and are presented relative to those in si-NT-TTs (**H**). Results are means ± SEM (*n* = 3); *p*: * ≤ 0.05; ** ≤ 0.01; *** ≤ 0.001. Dashed line indicates the control level.

Since altered cancer metabolism could affect cell differentiation [31], as we indeed demonstrated for GBM [21], we examined the levels of several markers associated with differentiation of GBM (U-87MG), lung cancer (A549) and the triple negative breast cancer, ductal carcinoma (MDA-MB-231) cells in si-NT-TTs and si-hVDAC1-TTs (Figure 5).

In U-87MG cell-derived tumours, immunostaining for mature neuronal markers, including tubulin beta 3 (TUBB3) and glutamate decarboxylase 1 (GAD1/GAD-67), involved in GABA synthesis, revealed high expression levels in si-hVDAC1-TTs and low expression in the NT-TTs (Figure 5A,B), as reported previously [21]. Staining for glia fibrillary acidic protein (GFAP), expressed by astrocytes, was also observed in si-hVDAC1-TTs but to a lower degree in si-NT-TTs (Figure 5C). These results show that si-hVDAC1 treatment of U-87MG-derived tumours induced expression of neuronal markers, which suggests that tumour cells are differentiated into astrocyte- and neuron-like cells.

A549 cells are considered as not fully differentiated alveolar epithelial type II (AT2)-type cells [32]. We next asked whether these cells differentiated, for example, into mature AT2 or pulmonary alveolar type I (AT1) cells upon si-hVDAC1 treatment. In the lung, AT1 cells are long and thin flattened squamous cells that account for ~95% of the alveolar surface and lie adjacent to capillary endothelial cells to form the pulmonary gas exchange region. AT1 cells express aquaporin 5 (AQP5), homedomain-only protein x (HOPX) and podoplanin, also known as T1α or PDPN, a membranal mucin-type sialoglycoprotein. AT2 cuboidal surfactant-producing cells cover around 5% of the alveolar surface [33,34] and produce the pulmonary surfactant proteins (SP) A, B, C and D components of the surface-active lipoprotein complex, as well as the lipid transporter ABCA3, needed for proper lung function [35,36,37]. Increased IHC staining of SP-C was seen in si-VDAC1-TTs, relative to the staining level seen in si-NT-TTs (Figure 5D). q-RT-PCR analysis of SP-A1, SP-B and SP-D showed decreased or unchanged (e.g., SP-D) levels (Figure 5E). 

To identify AT1 cells, the mRNA expression levels of the AT1 markers podoplanin, AQP5 and HOPX was analysed by q-RT-PCR and were all found to be reduced (Figure 5E). These results suggest that the non-fully differentiated A549 cells further differentiated and that the cells in the si-hVDAC1-TTs are not identical to those in the si-NT-TTs. 

MDA-MB-231 human breast cancer cells correspond to a poorly differentiated triple negative breast cancer (TNBC) cell line that does not express the progesterone or estrogen receptors or the receptor tyrosine-protein kinase erbB-2 (ERBB2/Her2). To delineate the possibility of VDAC1 silencing driving MDA-MB-231 cells to differentiation, we analysed several markers proposed as reflecting differentiation [38]. CD44^+^/CD24^−^ breast cancer cells possess stem/progenitor cell properties [39]. CD44, a receptor of hyaluronan and other ligands, such as collagen types I and IV, as well as metalloproteinases of the extracellular matrix [33], are highly expressed in such cells. At the same time, the absence or low expression of CD24 by these cells was detected. In the si-hVDAC1-TTs, the levels of CD44 were down-regulated and those of CD24 were up-regulated, when evaluated by IF (Figure 5G). IF staining of Her2 showed increased expression levels in si-hVDAC1-TTs, relative to the expression noted in NT-TTs (Figure 5G). The expression levels of prolactin, estrogen and progesterone receptors (PRLR, ER, PR), and of Her2, CD24 and STAT5, associated with prolactin receptor activity, were all increased in si-hVDAC1-TTs, as analysed by q-RT-PCR (Figure 5H). 

These results demonstrate that si-hVDAC1 treatment of tumours derived from the three cell lines altered the expression of certain genes/proteins associated with CSCs or differentiation. 

### 3.5. VDAC1 Depletion Alters the Expression of a Master Transcription Factor (TF) 

**Figure 6:**
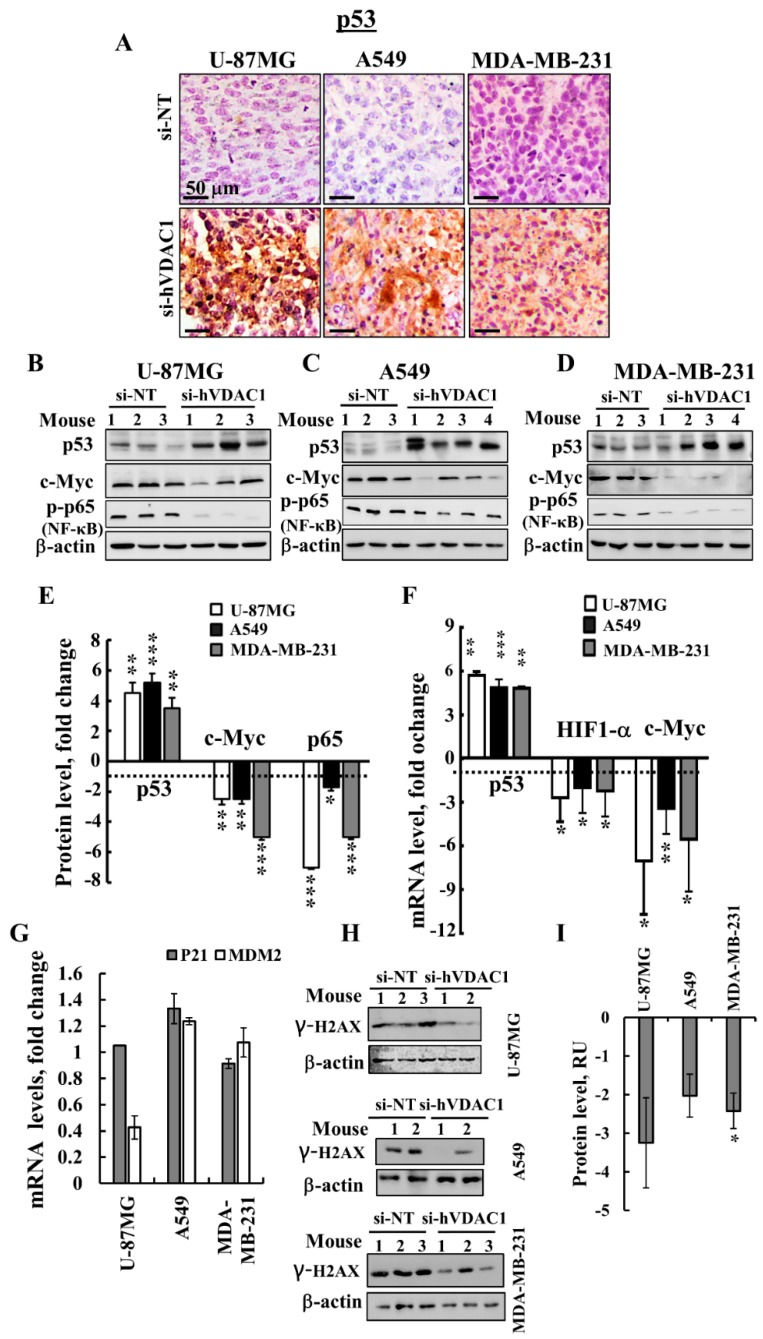
si-hVDAC1 treatment alters the expression levels of p53, HIF-1α and c-Myc transcription factors and of phosphorylated NF-κB/RelA, p65 in U-87MG-, A549- and MDA-MB-231-derived tumours. (**A**) Representative IHC staining of p53 in si-NT-TT or si-hVDAC1-TT sections derived from U-87MG, A549 and MDA-MB-231 xenografts. (**B**–**E**) Immunoblot analysis of p53, c-Myc and phosphorylated NF-κB/RelA (p65) in si-NT-TTs and si-hVDAC1-TTs derived from U-87MG (**B**), A549 (**C**) and MDA-MB-231 (**D**) xenografts. Quantitative analysis of p53, c-Myc and p65 levels in U-87MG, A549 and MDA-MB-231 cell tumours is presented (**E**). (**F**) q-RT-PCR analysis of p53, HIF-1α and c-Myc mRNA levels in U-87MG-, A549- and MDA-MB-231-derived tumours treated with si-hVDAC1, relative to those treated with si-NT. (**G**) q-RT-PCR analysis of p21 and MDM2 mRNA levels in U-87MG-, A549- and MDA-MB-231-derived tumours treated with si-hVDAC1, relative to those treated with si-NT. (**H**,**I**) Immunoblot staining (**H**) of γ-H2AX (phospho S139) using specific antibodies and their quantitative analysis **(I)** presented as relative units (RU); mean ± SEM (*n* = 3 mice) are shown for si-NT-TTs or si-hVDAC1-TTs derived from U-87MG, A549 and MDA-MB-231 xenografts. Results are means ± SEM); *p*: * ≤ 0.05; ** ≤ 0.01; *** ≤ 0.001. Dashed line indicates the control level.

For a better understanding of the molecular mechanism underlying cell signalling and gene expression alteration by si-hVDAC1 tumour treatment, we assessed the expression levels of the canonical major TFs p53, HIF-1α (hypoxia-inducible factor 1 alpha) and c-Myc, which regulate metabolism, cell growth, proliferation and differentiation [40]. si-hVDAC1 tumour treatment resulted in an elevation (5-6-fold) of p53 levels in tumours derived from U-87MG, A549 or MDA-MB-231 (expressing mutated p53) cells, while the levels of expression of HIF-1α (Figure 6A–F) and c-Myc (Figure 6B–F) were reduced, as revealed by IHC, immunoblotting and q-RT-PCR analyses. 

Although U-87MG and A549 cells express wild-type p53, while MDA-MB-231 cells express mutated p53, p53 was up-regulated in the three cell lines. We further evaluated the relationship between si-VDAC1-induced increases in p53 levels and those of MDM2 and p21 (WAF1) (Figure 6G). mRNA levels of the cyclin-dependent kinase (CDK) inhibitor p21 (WAF1/CIP1; CDKN1a) [41] were not significantly changed in the three cell lines, while mRNA levels of MDM2, which induces ubiquitin-dependent degradation of [42,43] were decreased in the si-hVDAC1-treated GBM tumours, but not in A549 or MDA-MB-231–derived xenografts (Figure 6G). 

To test whether the increased p53 levels is associated with damaged chromosomal DNA, we analysed the expression levels of γ-H2AX (phospho S139) in sections derived from U-87MG, A549 and MDA-MB-231 xenografts by immunobloting using specific antibodies (Figure 6H). As shown the levels of γ-H2AX were reduced in the in si-hVDAC1-TTs derived from U-87MG, A549 or MDA-MB-231 cells.

Finally, we analyzed the expression levels of the master regulator nuclear factor-light-chain-enhancer of activated B cells (NF-κB/RelA (p65), responsible for coordinating many of the signals that drive inflammation, proliferation and oncogenesis, in si-NT-TTs and si-hVDAC1-TTs [44]. The level of phosphorylated NF-kB/p65 was highly decreased in si-hVDAC1-TTs derived from U-87MG, A549 or MDA-MB-231 cells (Figure 6B–F).

**Figure 7:**
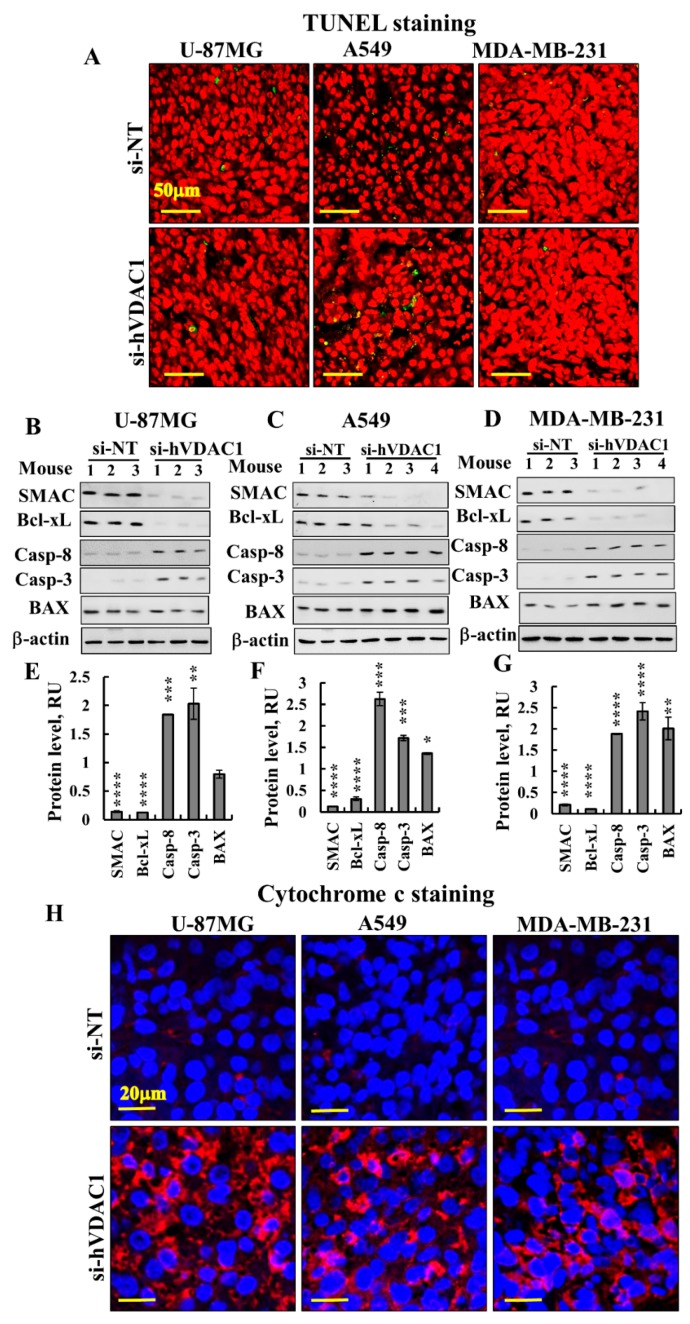
si-hVDAC1 treatment alters the expression levels of SMAC, Bcl-xL, BAX, caspase 8, caspase 3 and cytochrome *c* in U-87MG-, A549- and MDA-MB-231-derived tumours. (**A**) Representative sections of TUNEL staining of si-NT-TT or si-hVDAC1-TT sections derived from U-87MG, A549 and MDA-MB-231 xenografts. Immunoblots (**B**–**D**) of SMAC, Bcl-xL, caspase 8, caspase 3 and BAX and their quantitative analysis (**E**–**G**) in si-NT-TTs and si-hVDAC1-TTs derived from U-87MG (**B**, **E**), A549 (**C**, **F**) and MDA-MB-231 (**D**, **G**) xenografts. Average relative units (RUs) and β-actin as an internal loading control are shown. Results are means ± SEM (*n* = 3); *p*: ** ≤ 0.01; *** ≤ 0.001; **** ≤ 0.0001. (**H**) Representative cytochrome c staining of si-NT-TT and si-hVDAC1-TT sections derived from U-87MG, A549 and MDA-MB-231 xenografts.

As p53 expression levels were highly increased, we examined whether si-hVDAC1 tumour treatment induced apoptosis. TUNEL staining for the visualisation of apoptotic cells showed no significant apoptosis in si-NT-TTs or si-hVDAC1-TTs derived from U-87MG, A549 or MDA-MB-231 cells (Figure 7A). The expression levels of pro-apoptotic SMAC, shown to possess non-apoptotic functions [45], as well as the anti-apoptotic protein Bcl-xL, were decreased in si-hVDAC1-TTs, while Bax levels were unchanged or increased in the three types of tumours (Figure 7B–G). 

Unexpectedly, the levels of expression of the pro-apoptotic proteins caspases 3 and 8 rose in si-hVDAC1-TTs (Figure 7B–G). Similarly, cytochrome c levels were highly increased in the three types of tumours, as demonstrated by IF staining (Figure 7H). Caspases 3 and 8 have been proposed to possess additional non-apoptotic functions, including those related to bioenergetics, differentiation, inflammation and metabolism [46,47,48,49,50]. Cytochrome c has been associated with the remodelling of nuclear chromatin and cell differentiation [51,52].

## 4. Discussion

**Figure 8:**
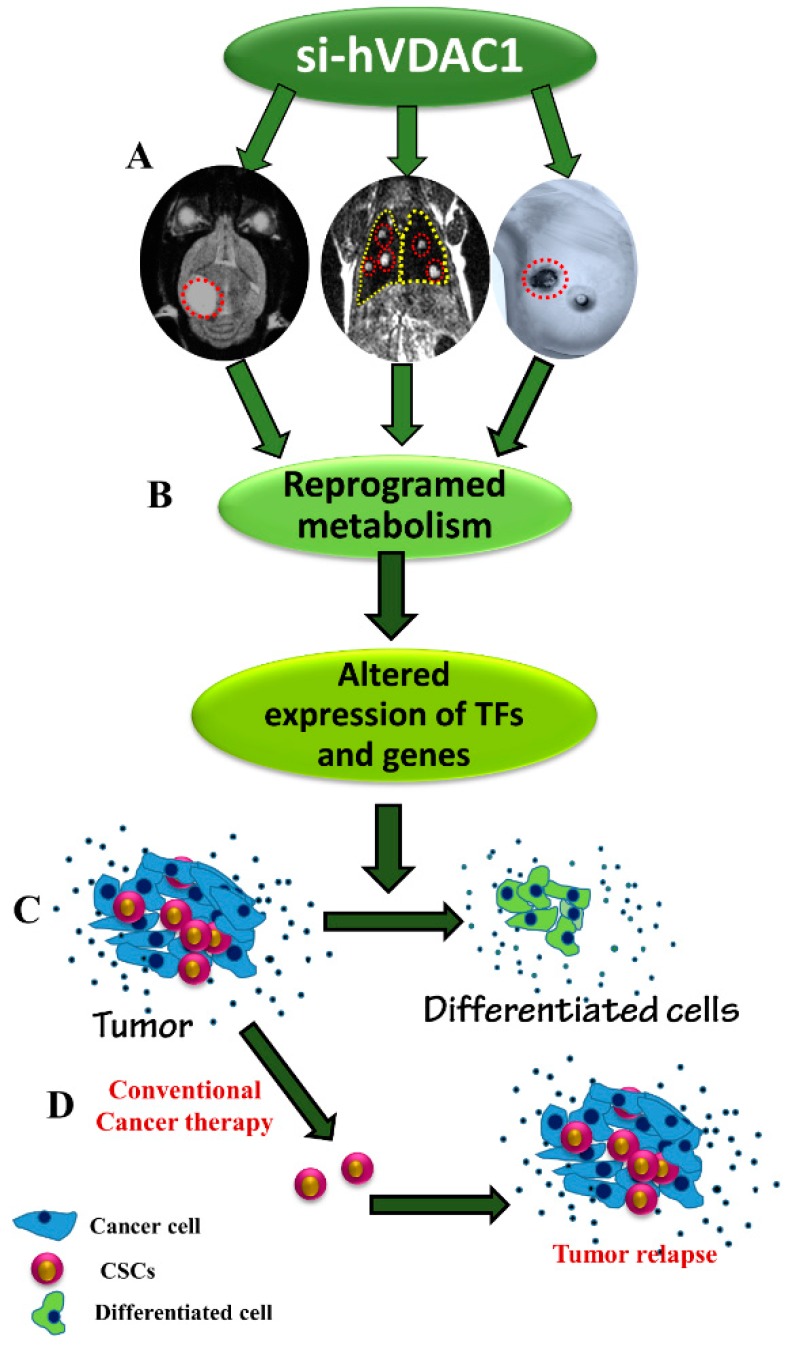
A schematic presentation of cancer cell VDAC1-depletion and metabolic reprogramming leading to a reversal of oncogenic properties and cell differentiation in U-87MG-, A549- and MDA-MB-231-derived tumours. The homeostatic energy and metabolic states of cancer cells in the tested U-87MG, A549 and MDA-MB-231 xenografts are modified upon silencing VDAC1 expression, leading to a reprogramming of metabolism, thereby decreasing energy and metabolite generation (**A**). This leads to changes in the levels of the master metabolism regulator p53, as well as HIF1-α and c-Myc expression levels, and of other genes (**B**), resulting in a reversal of oncogenic properties, including elimination of CSCs while leading to cell differentiation (**C**), thereby preventing tumour recurrence. Tumour treatment with conventional therapy, such as chemotherapy or radiation, targets cancer cells but not cancer stem cells (**D**). As such, tumour relapse may occur.

In this study, we used siRNA specific to human VDAC1 for treating GBM-, lung- and breast-derived subcutaneous tumours. Our results show that VDAC1 depletion caused a reversal of tumour-reprogramed metabolism that assaulted critical functional nodes in the oncogenic network, including arrested cell proliferation, and a reversal of cell properties to the non-oncogenic status, irrespective of the mutations carried. Remarkably, we also targeted cancer stem cells in the three tumour types and induced differentiation. As summarised in Figure 8, we suggest that reduced metabolism and energy production in the cancer cell induces an altered transcriptional program to control the revised energy homeostasis, thereby rewiring pathways for growth and survival underlying the malignant phenotype. Our findings point to VDAC1 as a significant control point for reprogramming metabolism, reversing the properties of cancer cells and thus, representing an emerging cancer drug target.

### 4.1. Reprogrammed Cancer Cell Metabolism is Reversed by VDAC1 Depletion, Resulting in Inhibited Cell Proliferation and Tumour Growth 

Cancer cells exhibit a variety of adaptive responses to stress, including angiogenesis, abnormal metabolic changes and de-differentiation. In terms of metabolic reprograming, aerobic glycolysis and enhanced glutamine utilisation are prime examples of such rewiring [53]. While cancer cell metabolism is a promising target [54,55,56], targeting metabolism is difficult because of the nature of metabolic plasticity, adaptation and redundancy. Here, we present the novel concept for modulating the metabolism of cancer cell by depleting VDAC1, a protein that has a central role in cell energy and metabolism [14,15,16] and which is over-expressed in many tumours, including GBM, lung and breast cancers (Figure 1A) [16,21]. Upon VDAC1 depletion, cancer cell energy and metabolic homeostasis are impaired [16,21] (Figure 3), leading to de-programming of cancer cell energy and metabolism, involving changes in the expression of the TFs and genes associated with metabolic regulation (Figure 6).

Concomitant with the notable decrease in VDAC1 expression levels, the growth of tumours derived from lung or breast cancer or GBM cells was inhibited (Figure 2). The residual si-hVDAC1-TTs demonstrated a massive reduction in the expression of Glut-1, HK-I, GAPDH and LDH-A (Figure 3, Figure S1A), fostering enhanced aerobic glycolysis in the untreated tumour. VDAC1 depletion, however, also resulted in decreased levels of expression of Kreb’s cycle and OXPHOS enzymes (i.e., CS, complex IVc and ATP synthase 5a) (Figure 3, Figure S1A). This agrees with studies suggesting that cancer cells maintain significant OXPHOS capacity and can switch from glycolysis to OXPHOS during carcinogenesis, based on the prevailing normoxic or hypoxic environmental conditions [4,57]. 

Thus, VDAC 1 silencing decreased all metabolism-related processes, including glycolysis, TCA cycle and OXPHOS, as no substrates for these pathways were available due to the absence of VDAC1, which acts as a transporter for substrates in and out of the mitochondria. 

The response of cancer cell energy demands is controlled by AMPK, whose activity goes up when metabolic stress conditions are induced by restriction of cellular ATP [27]. Our results showed that in the three cancers considered, the high levels of activated p-AMPK in si-NT-TTs reduced significantly in si-hVDAC1-TTs (Figure 3J–L). Indeed, AMPK was demonstrated to be activated hugely in vivo in human and rodent glioblastomas [58]. Moreover, activation of AMPK can be a pro-tumourigenic signal in cancer and hence a possible therapeutic target in cancer treatment [26]. Furthermore, AMPK regulates many transcription factors, their co-activators, and histones to stabilise gene expression and nuclear events, which leads to cell survival and metabolic reprogramming [27]. This agrees with the rewired metabolism as induced by VDAC1 depletion.

Another signalling pathway associated with energy-associated cancer cell growth and survival is the mTOR pathway that senses cellular energy, oxygen levels, and nutrient to stabilise cell growth and survival [29]. The levels of mTOR and its downstream effector phosphorylated S6 [30] declined significantly in si-hVDAC1-TTs (Figure 3J–L).

In summary, in tumours derived from GBM, breast and lung cancers, regardless of the cellular origin or mutations carried, VDAC1 depletion led to similar metabolic reprogramming, including reversal of the cancer cell’s metabolic adaptation as controlled by the AMPK and mTOR signalling pathways. 

### 4.2. Alterations in the Expression of TFs are Involved in si-hVDAC1-Induced Reprogramming of Cancer Cells 

The overall process of cell metabolism is tightly controlled by transcriptional regulation at the DNA, RNA, post-translational modification and epigenetic levels. We showed here that reprogramming metabolism via silencing VDAC1 expression affects the expression of key transcription factors in GBM, lung and breast cancers. p53, HIF-1α and c-Myc regulate metabolism, cell growth, proliferation and differentiation, with the interplay between these master TFs, mediated via other TFs and genes, regulating the ‘transformed phenotype’ [40]. In the three tumour types tested here, p53 expression levels were highly increased, while the levels of HIF-1α and c-Myc declined in si-hVDAC1-TTs (Figure 6). In addition, si-VDAC1 treatment reduced the levels of the phosphorylated NF-κB RelA/p65 subunit (Figure 6B–F). 

Under metabolic stress, p53 causes metabolic re-modelling and improves catabolism while coordinating a reduction in proliferation and cell growth [59]. Here, we induced metabolic stress in cancer cells by down-regulation of VDAC1, a scenario that led to increased expression of p53, with the outcome of cell-fate decisions being mediated by the transcription-dependent and -independent responses that regulate some aspects of cellular metabolism, thereby counteracting many of the metabolic changes that occur with cancer development [60]. 

While U-87MG and A549 cells express wild -type p53, MDA-MB-231 cells express mutated p53. All responded similarly to si-hVDAC1, showing elevated expression of p53 (4-6-fold increases). The markedly increased expression of p53 in si-hVDAC1-TTs is in accordance with the anti-tumourigenic activity of this TF, yet, apoptotic cell death was not recorded (Figure 7A). It is possible that p53 is deacetylated at lysine 382 by SIRT1, shown to have a decreased ability to cause apoptosis [61]. SIRT1 expression is elevated in si-hVDAC1-TTs (Figure 3J–L). In addition, the central roles of p53 in the regulation of the cell cycle by transcription-dependent and -independent mechanisms [59,62], as well as functions in CSC maintenance and differentiation, should be considered [31].

HIF-1α stabilises multiple adaptive responses to hypoxia, such as angiogenesis, proliferation and metabolism by coordinating the altered metabolic circuitry of the cancer cell, triggering the up-regulation of some genes that play a role in aerobic glycolysis [63]. HIF-1α also contributes to altered invasion, metastasis, stemness properties and disease relapse [64], as well as to the maintenance of CSC populations and genomic instability [63]. The decreased expression of HIF-1α in si-hVDAC1-TTs (Figure 6) should diminish these HIF-1α-induced pro-tumourigenic effects. In fact, several HIF-1α inhibitors that are in clinical trials (for example, against melanoma, breast cancer and GBM), used in combination with chemotherapy, result in increased tumour responsiveness to treatment and a reduced tumour progression rate [65,66].

Many growth-promoting signalling pathways and glucose metabolism genes (e.g., Glut-1, HK1, LDH-A) [67] are regulated by c-Myc, with LDH-A playing a key role in carcinogenesis [68]. The reduction in the levels of c-Myc in si-hVDAC1-TTs would thus antagonize these c-Myc-mediated pro-tumourigenic effects.

Finally, SIRT1, a NAD^+^-dependent deacetylase acting as a major metabolic/energy sensor that couples the cellular metabolic/energy status to transcriptional activity [69,70], is highly increased in the si-hVDAC1-TTs. It should be noted that SIRT1 plays diverse roles in cancer biology [71]. SIRT1 levels that were increased in si-hVDAC1-TTs (Figure 3J–L) can be associated with its function in metabolism regulation but also with a role in deacetylating p53 [61] or NF-κB. Levels of the phosphorylated RelA/p65 NF-κB subunit of NF-κB, were reduced upon si-VDAC1 treatment (Figure 6B–F). It has been shown that *Sirt1^+/−^/p53^+/−^* mice develop tumours in multiple tissues, whereas activation of SIRT1 by resveratrol treatment decreases tumourigenesis, suggesting that SIRT1 is a tumour suppressor [72]. SIRT1 also regulates NF-κB-regulated gene expression by deacetylating the RelA/p65 subunit, thereby inhibiting cell survival and NF-kappaB-dependent transcription [73].

In summary, the decreased expression of c-Myc, HIF-1α and the phosphorylated NF-κB- RelA/p65 subunit and P-AMPK, affecting related pathways as does the mTOR signalling pathway, and the increased expression of p53 in si-hVDAC1-TTs agree with their regulation of cancer cell metabolism. 

### 4.3. si-hVDAC1 Reduces CSC Levels and Induces Differentiation

Increasing evidence supports the hypothesis that tumour CSCs represent a subpopulation of malignant cells resistant to conventional cytotoxic/anti-proliferative therapies, constantly feeding the tumour with a supply of cancer cells [74,75,76]. CSCs were identified in many human malignancies, such as the brain, breast, pancreas, colon, ovary, and liver cancers, as well as leukaemia [77,78,79]. As CSCs are proposed to be involved in the growth of a tumour and are resistant to some chemotherapeutic strategies [80], and, moreover, obtain metastatic capacity [81], their targeting is of utmost importance. 

Here, we demonstrated that VDAC1 silencing highly reduced the expression of CSC markers in GBM and lung and breast cancer, as revealed using IHC, immunoblotting or q-RT-PCR (Figure 4, Figure S1B). We suggest that the disappearance of CSCs is associated with the metabolic reprogramming induced by silencing VDAC1 expression and the activation of an altered transcriptional program.

The presence of tumour glioma stem cells (GSCs) has been demonstrated [21,82,83]. We demonstrated that GSC markers, such as ALDH1, Nestin, SOX2, CD133, CD44, KLF4, Oct3/4 and Nanog, were highly decreased upon VDAC1 depletion (Figure 4, Figure S1B). CSC phenotypes in human lung cancer have also been defined [84,85]. Here, we showed that VDAC1 silencing in A549 cell-derived tumours resulted in reduced expression of lung-specific CSC markers [84,86], such as ALDH1, KLF4, SOX2, CD133, CD44, CD144, EPCAM, Oct3/4 and Nanog (Figure 4, Figure S1B). Breast cancer stem cell (BCSC)-specific markers [87] were highly reduced upon VDAC1 silencing in tumours established from the TNBC MDA-MB-231cell line, including ALDH1, CD133, EPCAM, SOX2 and KLF4 (Figure 4, Figure S1B).

As the dormant state of CSCs is considered to decrease their response to chemotherapy, novel approaches for targeting CSCs are required for the complete eradication of tumours [88]. Moreover, therapeutic approaches that target both CSCs and non-CSCs together would be the most effective cancer treatment. Thus, the targeting both CSCs and cancer cells by si-hVDAC1 treatment is a promising approach to make cancer more sensitive to irradiation and chemotherapy and may address any concerns about the tumour-forming potential of pluripotent stem cells [89]. Metabolic reprogramming of cancer cell via VDAC1 depletion highly reduced CSC markers, the result of inhibition of CSC proliferation and/or due to the induction of differentiation. Indeed, we demonstrated the inhibition of cell proliferation and induction of cell differentiation upon VDAC1 depletion.

Differentiation represents a process whereby an undifferentiated cell is transformed into a specialised differentiated cell. Here, we showed that following metabolism reprogramming, the residual “tumour” derived from the U-87MG cancer cell line that originated from astroglia showed up-regulated expression of neuronal markers, such as GFAP, TUBB3 and GAD-67, an enzyme involved in GABA synthesis (Figure 5A–C). Similarly, we showed that upon VDAC1 depletion in tumours derived from the A549 lung cancer cell line, representing non-mature AT2 cells, the residual tumour showed increased expression of the pulmonary-associated surfactant protein C, SP-C, although SP-A1 and SP-B levels were decreased (Figure 5D,E). In addition, AT1 cells were revealed by the presence of markers such as podoplanin, AQP5 and HOPX, which were reduced in the si-hVDAC1-TTs (Figure 5E). 

We expected increased expression of podoplanin in that case where AT2 cells had differentiated into AT1 cells. The decreased PDPN expression in si-hVDAC1-TTs can be related to PDPN presence on the surface of many types of normal cells, such as endothelial cells in lymphatic vessels, and not only in AT1 cells [90]. PDPN is often up-regulated in cancer, particularly in squamous cell carcinomas, such as cervical, skin and lung cancers [90,91] and plays a key role in cancer cell invasiveness by controlling invadopodia [92]. PDPN expresses in various tumours during migration and metastasis [91]. Thus, by decreasing PDPN expression, si-hVDAC1 countered the pro-cancer activity of PDPN in terms of cancer progression and metastasis. 

These results suggest that non-fully differentiated A549 cells within the si-hVDAC1-TTs are not identical to those in the si-NT-TTs and may represent further differentiation into less malignant lineage states.

Finally, in the residual tumour of a TNBC cell line-based MDA-MB-231-derived tumour following si-hVDAC1 treatment, increased levels of prolactin, estrogen and progesterone receptors, as well as Her2, were noted (Figure 6G,H). TNBC is a form of breast cancer that is highly malignant and has poor prognosis. TNBC does not respond to endocrine therapy and often defies current chemotherapeutic agents. Therefore, the results presented here suggest that si-hVDAC1 could, for example, allow the use of herceptin (anti-Her2-antibodies) as an effective therapeutic agent for TNBC patients. The levels of CD24 and STAT5, which is a mammary gland factor associated with prolactin receptor activity [93], were also augmented, suggesting differentiation into less malignant lineages.

This reprogrammed metabolism-induced differentiation in the three types of cancer may involve the down-regulation of CSC TFs, such as OCT3/4, Nanog and SOX2. Indeed, these TFs orchestrate the gene regulatory network that supports self-renewal [94]. It has been shown that pluripotency can be induced in somatic cells by the over-expression of OCT3/4, SOX2, KLF4, and c-Myc [95]. All were much reduced in the si-hVDAC1-TTs (Figure 4). Tumour cells undergoing differentiation upon metabolic reprogramming is also reflected in the over-expression of other proteins associated with differentiation, such as the increased expression of p53, cytochrome c, and caspases 3 and 8 [46,47,48,49,50] (Figure 6 and Figure 7).

The increase in p53 expression levels in U-87MG derived tumours by si-VDAC1 may result from the down-regulation of MDM2 thereby decreasing ubiquitin-dependent p53 degradation. However, no such results were obtained with the A549- or MDA-MB-231-derived tumours (Figure 6G). The increase in p53 levels may be associated with chromosomal DNA damage. However, histone γ-H2AX expression levels were reduced (Figure 6H,I), suggesting that in the si-hVDAC1-treated residual “tumours”, chromosomal DNA damage was reduced. 

Considering that p53 levels were increased, that CDK2 and CDK4 levels were decreased at the protein level (Table 1), and that p21 levels were unchanged, we suggest that the enhanced p53 expression seen in si-hVDAC1- treated residual tumours is associated with cell differentiation, whether by cells expressing native ( i.e., U-87MG and A549 cells) or mutated p53 (i.e., MDA-MB-231 cells), via mechanisms independent of cell death [31]. The differentiation of mouse embryonic stem cells was induced by p53 via the suppression of Nanog expression [96] that was also found to be down-regulated in si-hVDAC1-TTs. As with U-87MG cells [21,97], an increase in cytochrome c levels was found in si-hVDAC1-TTs derived from A-549 and MDA-MB-231 cells without subsequent apoptosis. This relates to the role of cytochrome c in promoting cell differentiation and in the remodelling of chromatin in the nucleus [51]. The notable increases in caspases 3 and 8 levels in si-hVDAC1-TTs, again without apoptosis, also seems to be related to the additional role of these proteins in cell differentiation. Caspase 3 is necessary for differentiation [49,98] and contributes to tumourigenic transformation by interfering with cell differentiation [47]. Several cancer types were found with caspase 8 deficiency, with such deficiency playing a role in tumourigenic transformation by interfering with cell differentiation [47] Therefore, the over-expression of p53, cytochrome c and caspases in si-hVDAC1-TTs may reflect their non-apoptotic functions in the promotion of cell differentiation [97]. 

Together, the data presented here support the suggestion that VDAC1 depletion in cancer cells leads to metabolic reprogramming, which via different molecular processes mediated by TFs and epigenetics, directed the tumour cells to more differentiated stages. The differentiated cells can be CSCs, as their levels were profoundly decreased and are capable of in vivo differentiation toward less malignant lineages. Cell differentiation into a stage that cannot replicate would prevent tumour re-growth and relapse (Figure 8).

In conclusion, as summarised in Figure 8, we have demonstrated that VDAC1 depletion in GBM, lung cancer and TNBC led to a rewiring of cancer cell metabolism, resulting in an assault on crucially useful nodes in the oncogenic network. The findings of this study demonstrate that VDAC1 regulates a continuum of cellular functions, including metabolism and cell differentiation. Furthermore, the specific transcriptional networks established in the cancer cell could be altered via reprogramed metabolism, leading to the regulation of gene transcription. We showed that the rewired cancer cell energy and metabolism via VDAC1 depletion changed the expression of p53, c-Myc and HIF-1a, NF-kB/RelA and probably other TFs, antagonised their pro-growth functions, reduced the proliferation of cells and promoted differentiation. The reduced CSC levels may reflect their differentiation into less malignant lineages. Thus, by focusing on a single target, the mitochondria gatekeeper VDAC1, cancer-reprogrammed metabolism was reversed, leading to inhibited tumour growth in the three cancers tested. The ability of si-hVDAC1 to simultaneously attack several tumourigenic-associated processes offers a powerful and innovative therapeutic strategy. 

## 5. Conclusions

Cancer cell metabolism in culture or in tumour can be targeted by reducing the expression of the mitochondrial gatekeeper VDAC1 using specific siRNA, reversing cellular oncogenic properties. 

Treatment of mouse xenografts derived from glioblastoma (U-87MG cells), lung cancer (A549 cells) or triple negative breast cancer (MDA-MB-231 cells) by si-hVDAC1 resulted in reprogramed metabolism, decreased proliferation, inhibited stemness, altered expression of genes and proteins, and induction of cell differentiation toward less malignant lineages.

Such rewiring of cancer cell metabolism also targets cancer stem cells in breast and lung cancers and glioblastoma, with differentiation being induced regardless of cancer origin or mutational status.

Thus, VDAC1, regulating a continuum of cellular functions from metabolism to cell differentiation, offers a target for treating GBM, lung and breast cancer via attack on the interplay between metabolism and oncogenic signaling networks.

## Figures and Tables

**Table 1:** Cell cycle-related genes differentially expressed in U-87MG-derived tumours treated with si-NT or si-hVDAC1, as identified by DNA microarray analysis.

Gene	Fold of Change (*p*-Value)	Function
CDK1- Cyclin-dependent kinase 1 [HGNC:1722]	−7.5 (0.0081)	Essential for the G1/S transition in cell cycle progression and leads to preparation for S phase entry
CDK2- Cyclin-dependent kinase 2 [HGNC:1771]	−4.33 (0.0080)	Involved in the activation of proliferative TF and interacts with p21
CDK4- Cyclin-dependent kinase 4 [HGNC:1773]	−2.18 (0.016)	In neural stem cells, proposed to inhibit neurogenesis and expand the population of basal progenitors by shortening the duration of G1
Cyclin B1- G2/mitotic-specific cyclin-B1 [HGNC:1579]	−4.62 (0.0019)	Involved in regulating G2/M phase of the cell cycle. Contributes to the switch-like all or none behaviour of the cell in deciding to commit to mitosis
Cyclin B2- G2/mitotic-specific cyclin-B2 [HGNC:1580]	−5.51 (0.0127)	Play a key role in transforming growth factor beta-mediated cell cycle control
Cyclin D3- G1/S-specific cyclin-D3 [HGNC:1585]	3.64 (0.015)	Postulated to carry out cell cycle-independent functions in a range of terminally differentiated cells
CDK 5, regulatory subunit 1 (p35) [HGNC:1775]	6.12 (0.0035)	Cyclin 5 complex that is essential for neuronal synaptic activity
CDK 5, regulatory subunit 2 (p39) [HGNC:1776]	5.72 (0.016)	Involved in the activation of CDK5/TPKII

Selected genes associated with cell cycle or/and neuronal differentiation, are presented. Changed gene levels by ≥2-fold and a false discovery rate < 0.05 were used. For each gene, the gene symbol and name, linear fold of change in expression and *p*-value are indicated. Negative numbers represent down-regulation.

## Supplementary Materials

The following are available online at http://www.mdpi.com/2072-6694/10/12/499/s1, Table S1: Characterization of cancer cell lines used in this study, Table S2: Antibodies used in this study. Antibodies against the indicated protein, their catalogue number, source, and the dilutions used in IHC, immunoblot and immunofluorescence experiments are presented, Table S3: Real-Time PCR primers used in this study. The genes examined, and the forward and reverse sequences of the primers used are indicated, Figure S1: si-hVDAC1 treatment reduced expression of metabolism related (A) and cancer stem cell related (B) proteins in U-87MG-, A549- and MDA-MB-231-derived tumours.
