# Supplementary material for: Mitochondrial VDAC1 Silencing Leads to Metabolic Rewiring and the Reprogramming of Tumour Cells into Advanced Differentiated States
Source: Cancers (Basel). 2018 Dec 8;10(12):499. doi: 10.3390/cancers10120499 (PMC6316808; doi:10.3390/cancers10120499)
Supplement: Supplementary file 1 [file cancers-10-00499-s001.pdf]

cancers

Supplementary Data: Mitochondrial VDAC1 Silencing Leads to Metabolic Rewiring and the Reprogramming of Tumour Cells into Advanced Differentiated States

Tasleem Arif, Avijit Paul, Yakov Krelin, Anna Shteinfer-Kuzmine and Varda Shoshan-Barmatz

Table S1. Characterization of cancer cell lines used in this study.

Cell Line	Cancer Type	Origin	Mutations	Tissue Developed From	Reference
MDA-MB-231, breast adenocarcinoma aneuploidy cells	Breast, triple negative	Pleural effusion metastatic tumor	BRAF, RAS, CDKN2A, TP53, PTEN, BRIP1, LIFR	Epithelia	[1,2]
A549, carcinoma hypotriploid cells	Non-small cells lung	Primary tumor	RAS, CDKN2A, FLT3, CBL, KEAP1, ZFHX3, FH, FUS, STK11, ATR, SUFU, HIP1, SMARCA4	Epithelia	[1,2]
U-87MG, glioblastoma hypodiploid cells	GBM	Brain primary tumor	CDKN2A, RAS, PTEN, HF1, PCM1	Astrocytes/Glia	[1,2]

References:

1. ATCC cell lines. https://www.atcc.org/~/media/PDFs/Culture%20Guides /Cell_Lines_by_Gene_Mutation.ashx
2. Sites of cell lines mutations. http://cancer.sanger.ac.uk/cell_lines

Table S2. Antibodies used in this study. Antibodies against the indicated protein, their catalogue number, source, and the dilutions used in IHC, immunoblot and immunofluorescence experiments are presented.

Antibody	Source and Cat. No.	Dilution		
		IHC	WB	IF
Mouse monoclonal anti-ABCG2	GeneTex, Irvine, CA, cat: GTX60447	1:250	1:2000	-
Mouse monoclonal anti-β-actin	Millipore, Billerica, MA, MAB1501	-	1:10000	-
Rabbit monoclonal anti-ALDH1A1	Abcam, Cambridge, UK, ab52492	1:200	1:2000	-
Rabbit polyclonal anti-AMPK-p	Abcam, Cambridge, UK, ab23875	-	1:5000	-
Mouse monoclonal anti-ATPsyn. 5a	Abcam, Cambridge, UK, ab14748	1:300	1:1000	-
Rabbit polyclonal anti-BAX	Millipore, Billerica, MA, ABC11	-	1:2000	-
Bcl-xL		-	X	-
Rabbit monoclonal anti-pro-caspase 3	Abcam, Cambridge, UK, ab32150	-	1:2000	-
Rabbit monoclonal anti-caspase 8	Abcam, Cambridge, UK, ab108333		1:1000	
Rabbit polyclonal anti-CD144	Abcam, ab33168	1:50	-	-
Mouse monoclonal anti-CD24	Biolegend, San Diego, CA, cat: 311101	-	-	1:500
Rabbit polyclonal anti-CD44	Abcam, Cambridge, UK, ab157107	1:100	1:3000	-

Alexa Fluor 488 anti-mouse/human CD44	Biolegend, San Diego, CA, cat: 103015	-	-	1:500
Rabbit polyclonal anti-citrate synthase	Abcam, Cambridge, UK, ab96600	1:200	1:4000	-
Mouse monoclonal anti-c-Myc	Abcam, ab62928	-	1:10000	-
Mouse monoclonal anti-cytochrome *c*	BD Bioscience, San Jose, CA, 556432	-	-	1:250
Rabbit monoclonal cytochrome c oxidase subunit VIc	Abcam, Cambridge, UK, ab150422	1:200	1:2000	-
Mouse monoclonal anti-ERB B2/HER-2	Biolegend, San Diego, CA, cat: 324401	-	-	1:500
Mouse monoclonal anti-GAD-67	Abcam, Cambridge, UK, ab26116	-	-	1:1500
Mouse monoclonal anti-GAPDH	Abcam, Cambridge, UK, ab9484	1: 200	1:1000	-
Mouse monoclonal anti-GFAP	Santa Cruz Biotechnology, Inc. Dallas, TX, sc-33673	-	-	1:150
Rabbit monoclonal anti-Glut-1	Abcam, Cambridge, UK, ab40084	1: 200	1: 1500	-
Rabbit monoclonal anti-Ki-67	Thermo Scientific, NY, RM-9106-s1	1:100	-	-
Mouse monoclonal anti- γ-H2AX	Abcam, Cambridge, UK, ab26350	-	1:2000	-
Mouse monoclonal anti-HK-I	Abcam, Cambridge, UK, ab105213	1:500	1:2000	-
Rabbit polyclonal anti-KLF4	IMGENX Littleton, USA, IMG-6081-A	1:200	1:1000	-
Goat polyclonal anti-LDH-A	Santa Cruz Biotechnology, Inc. Dallas, TX, sc-27230	-	1:1500	-
Rabbit polyclonal anti-Nestin	Millipore, Billerica, MA, MAB353	1:400	1:1000	-
Mouse monoclonal anti-P53	Santa Cruz Biotechnology, Inc., Dallas, TX, sc-126	1:300	1:5000	-
Mouse monoclonal anti- p-NF-κB-p65	Santa Cruz Biotechnology, Inc. Dallas, TX, sc-135768	-	1:1000	-
Rabbit polyclonal anti-Prosurfactant protein C	Abcam, ab90716	1:250	-	-
Rabbit polyclonal anti-pS6	Cell Signalling Technology, Danvers, MA 01923, cat: 2215	-	1:1000	
Rabbit polyclonal anti-SIRT1	Millipore, Billerica, MA, cat:07-131	-	1:2000	-
Rabbit polyclonal anti-SMAC/Diablo	Abcam, Cambridge, UK, ab8115	-	1:2000	
Goat polyclonal anti-SOX2	Santa Cruz Biotechnology, Inc. Dallas, TX, sc-17320	1:200	1:1500	-
Mouse monoclonal anti-β III tubulin	Abcam, Cambridge, UK, ab7751	-	-	1:200
Rabbit monoclonal anti-VDAC1	Abcam, Cambridge, UK, ab154856	1:500	1:5000	-

Table S3. Real-Time PCR primers used in this study. The genes examined, and the forward and reverse sequences of the primers used are indicated.

Gene	Primer sequences
ß-Actin	Forward 5′-ACTCTTCCAGCCTTCCTTCC-3′<br>Reverse 5′-TGTTGGCGTACAGGTCTTTG-3′
ABCG2	Forward 5′-CACCTTATTGGCCTCAGGAA-3′<br>Reverse 5′-GAAACACTGGTTGGTCGTCA-3′
ALD1H1	Forward 5′-CCAAAGTCCTGGAGGTTGAA-3′<br>Reverse 5′-TAACTCCAGGCCATCACACA-3′
ATPsyn. 5a	Forward 5′-TCAGTCTACGCCGCACTTAC-3′<br>Reverse 5′-GACATCTCAGCAGTCCCACA-3′
AQP5	Forward 5′-GCCACCTTGTCGGAATCTAC-3′<br>Reverse 5′-CCCTACCCAGAAAACCCAGT-3′
CD24	Forward 5′-CCTGCAGTCAACAGCCAGT-3′<br>Reverse 5′-TTTTCCTTGCCACATTGGA-3′
CD133	Forward 5′-TGGGCTTGTCATAACAGGAT-3′<br>Reverse 5′-TTGCGGTAAAACTGGCTAAG-3′
c-Myc	Forward 5′-GTAGTGGAAAACCAGCAGCC-3′<br>Reverse 5′-CCTCCTCGTCGCAGTAGAAA-3′
CS	Forward 5′-AGGAACAGGTATCTTGGCTCT-3′<br>Reverse 5′-GGGGTGTAGATTGGTGGGAA-3′
ESR1	Forward 5′-TGGAGATCTTCGACATGCTG-3′<br>Reverse 5′-TCCAGAGACTTCAGGGTGCT-3′
GAPDH	Forward 5′-TGGAAGGACTCATGACCACA-3′<br>Reverse 5′-ATGATGTTCTGGAGAGCCCC-3′
GLUT1	Forward 5′-GGCCATCTTTTCTGTTGGGG-3′<br>Reverse 5′-TCAGCATTGAATTCCGCCG-3′
HER2	Forward 5′-ACAGTGGCATCTGTGAGCTG-3′<br>Reverse 5′-CCCACGTCCGTAGAAAGGTA-3′
EPCAM	Forward 5′-CTGGCCGTAAACTGCTTTGT-3′<br>Reverse 5′-TCCCAAGTTTTGAGCCATTC-3′
Hif-1α	Forward 5′-CTGACCCTGCACTCAATCAA-3′<br>Reverse 5′-TCCATCGGAAGGACTAGGTG-3′
HK-I	Forward 5′-GTCTCAGTCCAGCACGTTTG-3′<br>Reverse 5′-GAAACGCCGGGAATACTGTG-3′
HOPX	Forward 5′-TTAAGCAGGCTGGCATCAG-3′<br>Reverse 5′-TGCTCCGCTAGACCCTTCT-3′
Ki-67	Forward 5′-CTTTGGGTGCGACTTGACG-3′<br>Reverse 5′-GTCGACCCCGCTCCTTTT-3′
KLF4	Forward 5′-TGCCCCGAATAACCGCTG-3′<br>Reverse 5′-CGTTGAACTCCTCGGTCTCT-3′
LDH-A	Forward 5′-GCARabbitGGTGGTTGAGAGTGCTT-3′<br>Reverse 5′-GCACCCGCCTAAGATTCTTC-3′
MDM2	Forward 5′-GATCAGGATTCAGTTTCAGATCAGT-3′<br>Reverse 5′-CATCTGAGAGTTCTTGTCCTTCTTC-3′
Nanog	Forward 5′-TGGGATTTACAGGCGTGAGCCAC-3′<br>Reverse 5′-AAGCAAAGCCTCCCAATCCCAAAC-3′
Nestin	Forward 5′-GAAACAGCCATAGAGGGCAAA-3′<br>Reverse 5′-TGGTTTTCCAGAGTCTTCAGTGA-3′
Oct3/4	Forward 5′-GGGCTCTCCCATGCATTCAAAC-3′<br>Reverse 5′-CACCTTCCCTCCAACCAGTTGC-3′
P21	Forward 5′-CCAGCCTCTGGCATTAGAATTA-3′<br>Reverse -5′-CGGGATGAGGAGGCTTTAAATA-3′
p53	Forward 5′-AGGTTGGCTCTGACTGTACC-3′<br>Reverse 5′-AAAGCTGTTCCGTCCCAGTA-3′
Podoplanin	Forward 5′-ATCTGCCAACTTCAGAAAGCA-3′

	Reverse 5′-TTGTCTGTGTGTCTCCATCCA-3′
PR	Forward 5′-GTCTACCCGCCCTATCTCAAC-3′<br>Reverse 5′-ACCATAATGACAGCCTGATGC-3′
PRLR	Forward 5′-AATCTTGGCAGAGGCAGAAA-3′<br>Reverse 5′-TTTGGAGCTATTCCCATTGC-3′
SOX2	Forward 5′-CCATGCAGGTTGACACCGTTG-3′<br>Reverse 5′-TCGGCAGACTGATTCAAATAA-3′
SP-A1	Forward 5′-CTGGTCAGGCTCTCCATGA-3′<br>Reverse 5′-GCCCAGCTTAGACGTAGGC-3′
SP-B	Forward 5′-AGAGGTGCCATGGCTGAG-3′<br>Reverse 5′-CACAGGCCAAGGATGAGG-3′
SP-D	Forward 5′-GTTGAGGCCTTACAGGGACA-3′<br>Reverse 5′-CTGTGCCTCCGTAAATGGTT-3′
STAT5	Forward 5′-GTTGGTGGAAATGAGCTGGT-3′<br>Reverse 5′-AGGCTCTGCAAAAGCATTGT-3′
VDAC1	Forward 5′-AGACTGCAAAATCCCGAGTG-3′<br>Reverse 5′-CCAAACTCTGTCCCGTCATT-3′
VDAC2	Forward 5′-CGGCTACCAGATGAATTTTGA-3′<br>Reverse 5′-CTCTGTCCCGTCATTCACATT-3′
VDAC3	Forward 5′-AATAATGCCAGCCTGATTGG-3′<br>Reverse 5′-CTTGTGACCTCCTGCACTGA-3′

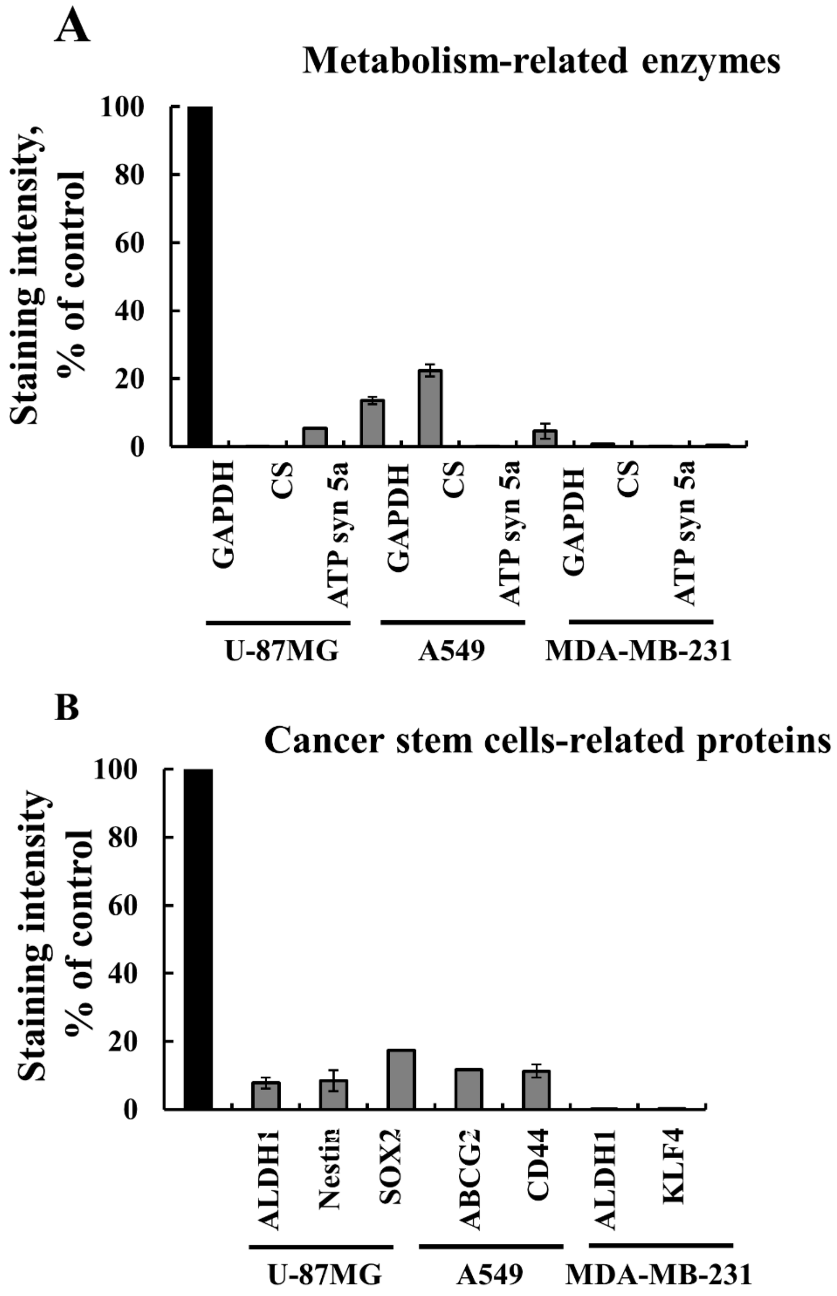

Figure 1S. si-hVDAC1 treatment reduced expression of metabolism related (**A**) and cancer stem cell related (**B**) proteins in U-87MG-, A549- and MDA-MB-231-derived tumours. Quantitative analysis of IHC stained si-NT-TTs or si-hVDAC1-TTs sections derived from U-87MG, A549 and MDA-MB-231 xenografts with specific antibodies against, GAPDH, CS, and ATP synthase 5a (ATP syn. 5a) (**A**) or against the indicated cancer stem cells markers. Quantitative analysis of the IHC images was carried out using a panoramic scanner (panoramic MIDI II, 3DHISTH) and HistoQuant software.
